# Depression and anxiety symptoms in male and female farmers: association with farm characteristics and mental health protection strategies in the FarmCoSwiss cohort

**DOI:** 10.1186/s12889-025-25407-z

**Published:** 2025-12-04

**Authors:** P. Ammann, A. Jeong, G. Lovison, J. Doetzer, S. Fuhrimann, M. Imboden, K. Ingold, M. S. Winkler, N. Probst-Hensch

**Affiliations:** 1 Swiss Tropical and Public Health Institute, Allschwil, Switzerland; 2 University of Basel, Basel, Switzerland; 3 Department of Environmental Social Sciences, Swiss Federal Institute of Aquatic Science and Technology, Überlandstrasse 133, Eawag, Dübendorf, 8600 Switzerland; 4 Institute of Political Science, University of Bern, Fabrikstrasse 8, Bern, 3012 Switzerland; 5 Oeschger Centre for Climate Change Research, University of Bern, Hochschulstrasse 4, Bern, 3012 Switzerland

**Keywords:** Agriculture, Cohort, Farmers, Mental health, Occupational health

## Abstract

**Background:**

Empirical research on farmers’ mental health in Switzerland is limited. This study uses cross-sectional data from the first follow-up of the *FarmCoSwiss* cohort to descriptively compare depression and anxiety symptom prevalence between Swiss farmers and the Swiss general population. Furthermore, the association of these mental health outcomes with farm-related characteristics and individual-based mental health protection strategies was explored.

**Methods:**

Of 878 adult baseline participants, 600 (68%, thereof 67% men) completed the follow-up survey between March and June 2024. Depression and anxiety symptoms were assessed using the Patient Health Questionnaire-9 (PHQ-9) and General Anxiety Disorder-7 (GAD-7) scales, respectively. Sum scores (0–27 and 0–21) were categorized as no/minimal, mild, moderate, or (moderately) severe symptoms for descriptive analyses. Key explanatory variables included five binary farm-related characteristics (farming system: non-organic or organic; production system: animal husbandry/mixed or no animal husbandry; farm size: ≤50 or > 50 hectares; label production: yes or no; management position: yes or no), and a binary indicator of the use of one or more individual-based mental health protection strategies (any or none). Depression and anxiety prevalences were descriptively compared to 2022 data from the Swiss Health Survey. Sex-stratified negative binomial regressions assessed associations between PHQ-9 and GAD-7 continuous sum scores and explanatory variables, adjusted for age, education, marital status, and season of participation.

**Results:**

Over half of the participants (52.3%) reported no depression and anxiety symptoms. Moderate or severe symptoms were observed in 10.7% (depression) and 9.5% (anxiety). Descriptive comparisons to the Swiss general population suggest that depression and anxiety symptoms are slightly more common among *FarmCoSwiss* participants. Women generally reported higher symptom prevalence. In men only, the use of any mental health protection strategy was associated with lower PHQ-9 and GAD-7 scores.

**Conclusions:**

While comparisons between two distinct study populations should be interpreted with caution, our findings suggest that depression and anxiety symptoms may be more prevalent among the Swiss farming population, particularly among women, than in the general population. Individual-based mental health protection strategies show promise for improving farmers’ mental health, but our results indicate a need for sex-specific strategies. Further longitudinal research on additional farm- and sex-specific factors affecting mental health in Swiss agriculture may be of interest.

**Supplementary Information:**

The online version contains supplementary material available at 10.1186/s12889-025-25407-z.

## Introduction

Research since the late 1980 s has repeatedly found high rates of poor mental wellbeing in farming communities in high-income countries [1, 2]. Cross-sectional surveys and cohort studies worldwide have found increased prevalences of depression in farmers as compared to non-farming working populations [3–5]. In addition, mental health conditions and occupational phenomena, such as depression, anxiety, or burnout, were identified as key contributing factors to reportedly high suicide rates in farming communities and linked to chronic work-related stress in agriculture [6–8]. From fluctuating commodity prices to animal disease outbreaks and environmental changes, farmers face a multitude of unpredictable conditions. The burden associated with such acute and chronic stressors may overwhelm farmers’ coping capacity and lead to mental health issues. Often cited risk factors for chronic stress include heavy workload and time pressure, institutional and economic demands, financial insecurity, social conflicts, and environmental and climate challenges [9–11]. Recent findings indicate that globalization, trade and financial deregulations, rural population decline, and climate and environmental disasters may even exacerbate farmers’ mental health challenges and suicide rates [2, 12, 13].

With regard to the association between farming systems and farmers’ mental health outcomes, some quantitative studies suggest a negative association between organic farming and depression, anxiety, and neurological symptoms as compared to conventional farmers, and higher life and job satisfaction in organic farmers [14–16]. However, other findings suggest no statistically significant differences regarding quality of life or depression between organic and conventional farmers [17–19]. There is no conclusive evidence indicating an association between farm-related characteristics, such as farm type or size, and farmers’ mental health [10, 20]. Some evidence indicates that animal husbandry and specialized production in particular are associated with poor mental health and depression due to higher exposure to risk factors such as high workloads, physically demanding work, regulatory pressure, and financial insecurity [21–23]. Other studies found no association between livestock farming and poor mental health in farmers [10, 24]. Farm owners and managers have been found to be more vulnerable to economic pressures and administrative burdens than farm employees [25]. However, farmers who identify more as entrepreneurs generally report higher work satisfaction [26]. Female farmers are often confronted with domestic and caring responsibilities while also being involved in off- and on-farm income generation [27]. Similarly, some quantitative and qualitative studies have reported higher stress and poorer mental health for women in agriculture as compared to their male colleagues [27–29]. Global evidence on mental health conditions in the general population shows similar prevalences in men and women, but sex-specific differences arise for specific diagnoses [30]. While prevalences of substance abuse and personality disorders are higher in men, women are more often diagnosed with anxiety and depression disorders [31].

In Switzerland, there are roughly 48,000 farms with an average size of 22 hectares (ha) and employing around 149,000 individuals in 2024 [32]. Research on Swiss farmers’ health in general and specifically mental wellbeing is scarce. A study by Reissig et al. suggests a higher burnout prevalence among Swiss farmers than in the general population [33]. They identified relational conflicts, financial struggles, heavy workload, a lack of free time, and self-reported bad physical health as predictors for burnout in both male and female farmers. While factors such as financial problems and time pressure were found to be predictors for burnout in both men and women, administrative responsibilities and a lower perceived relationship quality with their partner was related to burnout only in female farmers [33]. In addition, analysis of longitudinal data from the Swiss National Cohort SNC revealed an increased suicide rate in male farmers as compared to men of other professions, with a widening gap after 2006 [34]. While risk factors for this increased suicide rate in Swiss farmers have not been assessed in detail, possible explanations entail the stigma associated with seeking mental health support in agricultural communities and the impaired provision and utilization of mental health services in rural areas [35, 36]. Sex-specific analysis of suicide mortality rates in Switzerland points toward higher risk in male farmers as compared to their female colleagues [20, 37, 38]. Previous research within the *FarmCoSwiss* cohort (Swiss Farmer Family Health and Wellbeing Cohort) indicates that some farm-related characteristics, such as the farming system or farm size, are associated with human flourishing [39].

In view of the paucity of empirical evidence on farmers’ health in Switzerland, the first Swiss national farmer cohort *FarmCoSwiss* (www.swisstph.ch/farmcoswiss) was established in 2022 [40]. The cohort provides insights into the determinants, state, and (in the future) the temporal course of the physical and mental health and wellbeing of Swiss farmers and their partners. The present cross-sectional study analyzes data obtained during the cohort’s first follow-up survey with the objective to (i) descriptively compare the distribution of depression and anxiety symptoms between *FarmCoSwiss* participants and the Swiss general population, and (ii) explore the association of depression and anxiety symptoms with farm-related characteristics and individual-based mental health protection strategies in men and women. Potential associations of these variables with depression or anxiety symptoms may aid in identifying farming sub-populations at a higher risk of poor mental health and in need of targeted mental health protection strategies.

## Methods

The *FarmCoSwiss* cohort was established within the interdisciplinary Transformation in Pesticide Governance (TRAPEGO) project (www.trapego.ch) to investigate physical and mental health outcomes and occupational exposures in farmers and their partners [41]. In Chaps. 2.1 to 2.4, we will provide a summary of the cohort’s methods and describe the statistical analysis for the present study. Details on the objectives, sampling procedure, sample size, and methods employed in the *FarmCoSwiss* study are published elsewhere [40].

### Study participation

In 2022, the first recruitment round for the prospective *FarmCoSwiss* cohort was conducted. To participate in the study, individuals had to: (i) be at least 18 years old, (ii) speak one of the three national languages (German, French, or Italian), (iii) be employed in agriculture (full-time, part-time, or unpaid) or have a partner who works in agriculture, and (iv) be a long-term resident of Switzerland. Full- and part-time as well as unpaid individuals were recruited to reflect the shared occupational context and common stressors associated with agricultural work. Due to the lack of access to a national agricultural workforce registry and in view of the main objective of reducing loss to follow-up within the cohort, convenience sampling was used to recruit participants. A large distribution of print and digital flyers was part of the recruitment efforts, as were announcements at agricultural events, advertisements in agricultural periodicals, and newsletters from farmer associations and agricultural businesses. Self-registration of interested individuals took place through the web-based data collection and management tool REDCap (Research Electronic Data Capture), hosted by the Swiss Tropical and Public Health Institute [42, 43]. Upon registration, eligible participants were sent detailed study information and an informed consent form (ICF) by postal mail. To enhance participation rates and representativeness in terms of sex, age, and farming system, participant registration was monitored. Study invitations for the baseline survey were distributed between November 2022 and April 2023, and the online registration tool was accessible until August 2, 2023. In March 2024, participants who completed the baseline survey and agreed to be re-contacted for future studies received an invitation to the first follow-up survey.

### Data collection and variable overview

The online baseline questionnaire was sent to enrolled individuals via e-mail or, if requested, as a print version by postal mail in German, French, or Italian (see Supplement S1 for questionnaire translated into English). The survey included questions on participants’ socio-demographics including education and employment, farm management, farming system, agricultural production, and household structure.

Seven months after the closing of the baseline study enrolment, participants who had agreed to participate in future studies within the *FarmCoSwiss* cohort received an invitation to the first follow-up survey via e-mail or postal mail (see Supplement S2 for questionnaire translated into English). The follow-up questionnaire repeated selected questions from the baseline survey and additionally inquired about depression and anxiety symptoms, loneliness, individual-based physical and mental health protection strategies, and treatment obtained for physical or mental diseases or disorders.

At follow-up, the primary outcomes depression and anxiety symptoms were measured by the Patient Health Questionnaire-9 (PHQ-9) and the General Anxiety Disorder-7 (GAD-7) scales, respectively. The PHQ-9 assesses the degree of depression symptoms in an individual in the past two weeks and consists of nine questions with a scale ranging from 0 (not at all) to 3 (nearly every day) [44]. The total severity score ranges from 0 to 27, with higher scores indicating increased functional impairment and disability, and can be categorized into five severity levels (≤ 4: no/minimal, 5–9: mild, 10–14: moderate, 15–19: moderately severe, 20–27: severe) [44]. The GAD-7 entails seven items with a response scale from 0 (not at all) to 3 (nearly every day) and is used to screen for generalized anxiety disorder symptoms, panic disorder, social phobia, and post-traumatic stress disorder in the past two weeks [45]. Higher total severity scores indicate increased symptom-related difficulties, which can be classified into four categories (≤ 4: no/minimal, 5–9: mild, 10–14: moderate, 15–21: severe) [45]. Both tools are validated in primary care settings [46, 47]. The PHQ-9 has additionally been validated in occupational health settings [48].

 The primary explanatory variables of interest consisted of different farm-related and occupational characteristics, such as farming system (non-organic or organic), production system (animal husbandry/mixed production, no animal husbandry), farm size (≤ 50 ha, >50 ha), label production (yes or no), and having a farm management position (yes or no), as well as individual-based mental health protection strategies employed by farmers in their daily lives. Specifically, participants were asked how they, in general, protect their mental health in their everyday agricultural work. The multiple-choice answer options included: (i) acquiring knowledge (e.g., regarding stress management), (ii) working carefully (e.g., avoiding time pressure/stress), (iii) regular recovery/rest periods (e.g., hobby, time with friends/family), (iv) regular vacations, (v) regular physical activity, (vi) relaxation exercises (e.g., meditation, praying or writing a diary), (vii) talking to people of trust (e.g., friends), (viii) no active or conscious mental health protection. These answer options were chosen based on research investigating stress and mental health challenges in agricultural communities and in collaboration with farmers and agricultural experts [49, 50]. The final variable was binary (any or none). Participants with ambiguous answers (i.e., choosing at least one protection strategy and selecting that they do not actively or consciously protect their mental health) were allocated to the “no protection” category to obtain conservative model estimates. For the variable label production, participants were asked whether they sell at least some of their products under one or more food labels. They could choose from a list of more than 40 common food labels in Switzerland or/and add additional labels. The final variable was coded as label production ‘yes’ or ‘no’.

### Statistical analysis

Given that sex differences have been repeatedly reported for the prevalence of and risk factors for mental health outcomes, statistical analyses were by default stratified by sex [51]. Participants’ sex was assessed via self-report in the baseline questionnaire using a binary response option (“male” or “female”). This approach was chosen to ensure clarity and cultural appropriateness for the study population.

Descriptive analyses were employed to assess study population characteristics. Categorical variables are displayed as counts and percentages. Continuous variables are expressed as means and standard deviations (SD) or medians and ranges.

To better interpret the prevalences of depression and anxiety symptoms among *FarmCoSwiss* participants, frequencies in each PHQ-9 and GAD-7 category for men and women were descriptively compared to publicly available national PHQ-9 and GAD-7 data collected in the Swiss Health Survey (SHS) in 2022 [52, 53]. Every five years since 1992, the SHS collects population-representative data on health and wellbeing of the Swiss resident population aged 15 and older [54].

Given the right-skewed distribution of PHQ-9 and GAD-7 sum scores and the defined range between 0 and 27 (PHQ-9) and 0 and 21 (GAD-7), the association of the *PHQ-9* and *GAD-7 sum scores* (continuous) as endpoints with selected explanatory variables were explored using negative binomial regression models with log link. Due to the high correlation between PHQ-9 and GAD-7 scores (spearman *r* = 0.76), only the models for the PHQ-9 as the outcome variable are presented in the main text. Equivalent GAD-7 model results are displayed in the appendix.

The a priori selected explanatory variables included five farm-related characteristic variables (*farming system*: 0: non-organic, 1: organic; *production system*: 0: animal husbandry/mixed production, 1: no animal husbandry; *farm size* [ha]: 0: ≤50, 1: >50; *label production*: 0: yes, 1: no; *farm management*: 0: yes, 1: no), as well as the variable *mental health protection* (individual-based protection strategies employed: 0: any, 1: none).

The variables *age* (continuous), *highest education level* at follow-up (0: low/middle, 1: high), *marital status* (0: married, 1: single, 2: divorced/widowed), and *season* (when the follow-up survey was answered: before or after astronomical spring start on 20 March 2025, i.e. 0: cold or 1: warm season), were a priori included as confounders in all models.

In the primary analysis of interest, all primary explanatory variables and confounders were added to the PHQ-9 and GAD-7 models, respectively. Before running the regression models, collinearity between the explanatory variables was investigated by means of correlation matrices. Spearman correlation coefficients between explanatory variables were no higher than 0.33. Since missing values were below 3% for all variables of a priori interest, participants with missing data for the primary outcome, explanatory or confounder variables were excluded from the respective analysis (n_totalPHQ−9_ = 554, n_totalGAD−7_ = 555). As sensitivity analyses, participants with ambiguous answers in the mental health protection strategy variable (i.e., those who chose any protection strategy, but also “I do not actively or consciously protect my mental health”) were excluded from the primary (sex-stratified) PHQ-9 and GAD-7 models.

Regression coefficients with *p*-values below 0.05 were considered statistically significant. Statistical analyses were conducted in R version 4.3.3 using the *glm.nb* function with *log* link from the *MASS* (version 7.3–64) package for the negative binomial regression [55]. A simplified code for the regression analyses was uploaded on Zenodo (10.5281/zenodo.15359276).

### Ethical aspects

The *FarmCoSwiss* research project was performed in accordance with the current version of the Declaration of Helsinki, as well as all national legal and regulatory requirements (Human Research Act (HRA) and Human Research Ordinance (HRO)). Ethical approval for both the baseline and follow-up study was granted by the regional ethics committee, Ethikkommission Nordwest- und Zentralschweiz (EKNZ) (BASEC No. 2022 − 00549). All participants provided written informed consent prior to enrolment.

## Results

### Study participants’ characteristics

**Fig. 1:**
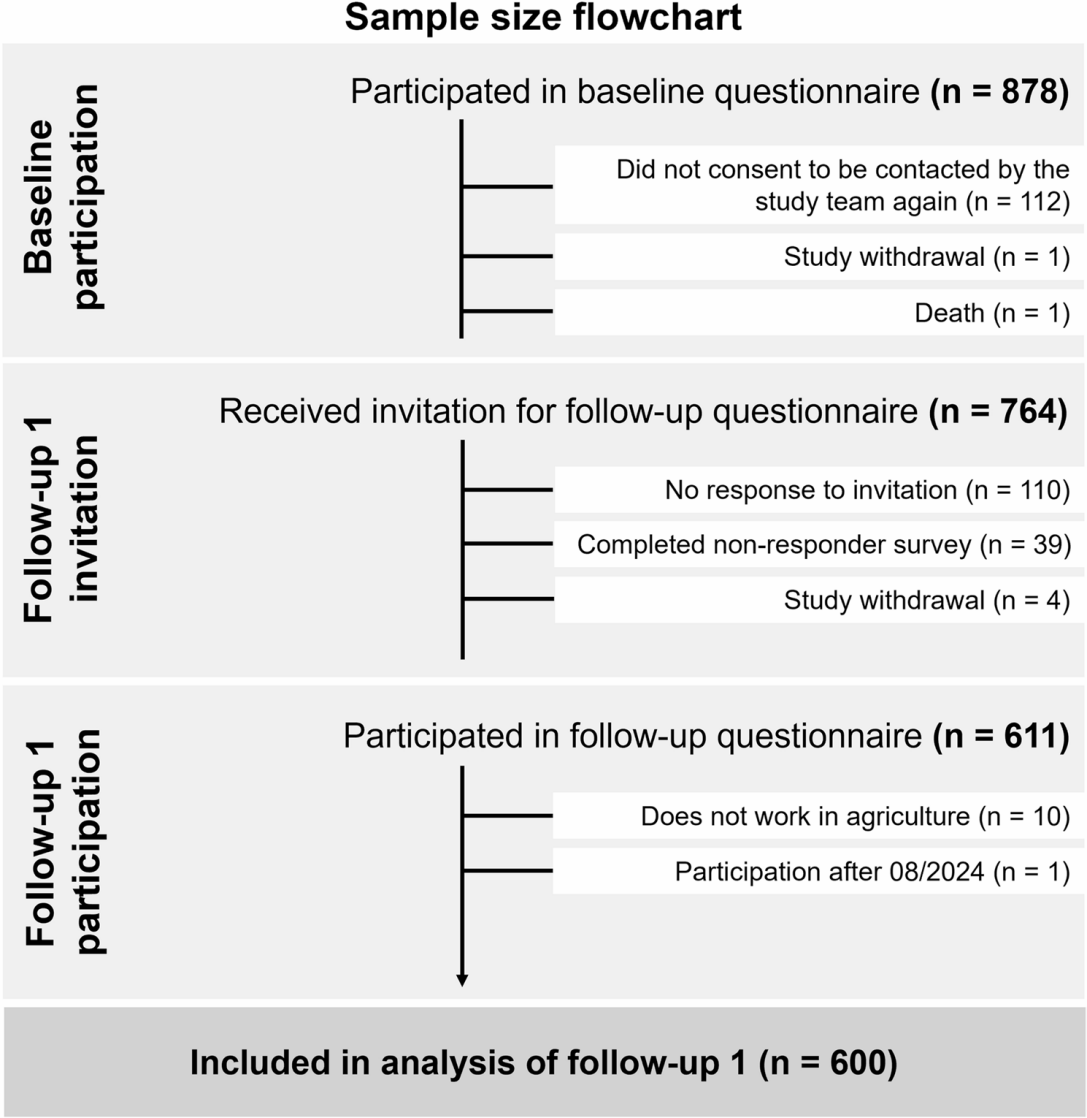
CONSORT flow chart of individuals participating in the baseline questionnaire, withdrawn or lost from the study, and included in data analysis of the first follow-up questionnaire in the *FarmCoSwiss* cohort.

The CONSORT flowchart (Fig. 1) depicts study participants’ progress since their participation in the baseline questionnaire. A flow diagram showing participants’ progress since recruitment is published elsewhere [40]. Of a total of 878 individuals filling out the baseline questionnaire, 764 individuals received an invitation to the first follow-up questionnaire, and 611 (80.0%) participated. Overall, data of 600 participants was included in the analysis for the present publication. The mean time difference between baseline and follow-up survey completion was 347 days (median: 341 days), with a minimum of 98 and a maximum of 536 days.

Participant characteristics at baseline comparing participants and non-participants in the first follow-up survey are displayed in Table A1 in the appendix. Slightly more participants with higher educational level and with animal husbandry participated at follow-up.

*FarmCoSwiss* study participants’ characteristics of farmers participating in the first follow-up survey are displayed in Table 1. Men made up two thirds of participants (*n* = 402, 67.0%) and the age range of the study sample at follow-up was 25 to 83 years. Half of participants reported a high educational level (*n* = 296, 49.3%) and the majority of study participants were married (*n* = 476, 79.3%). Most farmers worked on a non-organic farm (*n* = 462, 77.0%) and on farms with animal husbandry alone or in combination with crop production (*n* = 548, 91.3%). Roughly 90% of farms were smaller than or equal to 50 ha in size (*n* = 528, 88.0%). The majority of participants were farm (co-)managers (*n* = 436, 72.7%) and produced their goods under at least one agricultural label (*n* = 515, 85.8%). Compared to men (83.3%, *n* = 335), fewer women (51.0%, *n* = 101) were farm managers.

**Table 1:** *FarmCoSwiss* participant and farm-related characteristics at follow-up, stratified by sex

	Total	Men	Women
Sociodemographic variables			
Participants, n (%)	600 (100)	402 (67.0)	198 (33.0)
Age [years]			
mean (± SD)	49.9 (± 11.3)	51.3 (± 11.4)	47.1 (± 10.4)
min-max	25–83	25–83	27–74
Highest educational level ^a)^, n (%)			
low/middle	301 (50.2)	209 (52.0)	92 (46.5)
high	296 (49.3)	191 (47.5)	105 (53.0)
missing	3 (0.5)	2 (0.5)	1 (0.5)
Marital status			
married	476 (79.3)	318 (79.1)	158 (79.8)
single	82 (13.7)	52 (12.9)	30 (15.2)
divorced/widowed	37 (6.2)	29 (7.2)	8 (4.0)
missing	5 (0.8)	3 (0.8)	2 (1.0)
Farm-related characteristics			
Farming system, n (%)			
non-organic	462 (77.0)	309 (76.9)	153 (77.7)
organic	136 (22.7)	92 (22.9)	44 (22.3)
missing	2 (0.3)	1 (0.2)	1 (0.5)
Production system, n (%)			
animal husbandry ^b)^	548 (91.3)	366 (91.0)	182 (91.9)
no animal husbandry	48 (8.0)	33 (8.2)	15 (7.6)
missing	4 (0.7)	3 (0.8)	1 (0.5)
Farm size [hectares], n (%)			
≤ 50	528 (88.0)	347 (86.3)	181 (91.4)
> 50	71 (11.8)	54 (13.4)	17 (8.6)
missing	1 (0.2)	1 (0.2)	0 (0)
Farm management position, n (%)			
yes	436 (72.7)	335 (83.3)	101 (51.0)
no	164 (27.3)	67 (16.7)	97 (49.0)
Label production ^c)^, n (%)			
yes	515 (85.8)	352 (87.6)	163 (82.3)
no	67 (11.2)	43 (10.7)	24 (12.1)
missing	18 (3.0)	7 (1.7)	11 (5.6)

^b)^Includes any farm with animal husbandry, either with or without crop production

^c)^Participants were asked whether they sell at least some of their products under one or more food labels. They could choose from a list of more than 40 common food labels in Switzerland and/or add additional labels. The final variable was binary (label production: yes or no)

^a)^Education: low = mandatory primary and secondary education (≤9 years), middle = vocational training or high school (≤12 years), high = higher technical or vocational school or university (>12 years)

Study participants’ health-related characteristics are presented in Table 2. When asked if they had any physical or mental health protection strategy in place in their everyday agricultural work, roughly 90% (*n* = 533) of farmers had one or more protection strategies in place to protect their physical health, and almost 80% to protect their mental health (*n* = 480). Nearly half of individuals (*n* = 298, 49.7%) reported to have had a physical (*n* = 260, 43.3%) and/or a mental (*n* = 76, 12.7%) health issue in the past 12 months. While 81.5% of individuals with a self-reported physical health issue sought treatment, only 35.5% of mental health issues within the past 12 months were treated. Regarding depressive symptoms within the previous two weeks as measured by the PHQ-9, the majority of participants had no (*n* = 354, 59.0%) or mild symptoms (*n* = 177, 29.5%), and 10.7% reported at least moderate symptoms (*n* = 64). Most participants reported no (*n* = 396, 66.0%) or mild (*n* = 143, 23.8%) anxiety symptoms in the previous two weeks as measured by the GAD-7. Roughly 7% of individuals had an indication of severe anxiety symptoms (*n* = 40). Women more frequently reported using mental health protection strategies, having had a mental health issue in the past 12 months and having sought treatment for it, and exhibiting at least mild symptoms of depression or anxiety than men.

**Table 2:** *FarmCoSwiss* participants’ health characteristics, stratified by sex

Health variables			
Health protection strategy, *n* (%)			
Physical health			
any	533 (88.8)	352 (87.5)	181 (91.4)
none	52 (8.7)	40 (10.0)	12 (6.1)
missing	15 (2.5)	10 (2.5)	5 (2.5)
Mental health ^a)^			
any	480 (80.0)	306 (76.1)	174 (87.9)
none	105 (17.5)	86 (21.4)	19 (9.6)
missing	15 (2.5)	10 (2.5)	5 (2.5)
Self-reported health issue in past 12 months, n (%)			
yes	298 (49.7)	192 (48.7)	106 (53.6)
no	292 (48.6)	204 (50.8)	88 (44.4)
missing	10 (1.7)	6 (1.5)	4 (2.0)
Physical			
Total ^b)^	260 (43.3)	172 (42.8)	88 (44.4)
thereof treated	212 (81.5)	138 (80.2)	74 (84.1)
thereof untreated	48 (18.5)	34 (19.8)	14 (15.9)
Mental			
Total ^b)^	76 (12.7)	43 (10.8)	33 (16.8)
thereof treated	27 (35.5)	13 (30.2)	14 (42.4)
thereof untreated	49 (64.5)	30 (69.8)	19 (57.6)
PHQ-9 (degree of depression symptom severity) ^c)^, n (%)			
no/minimal	354 (59.0)	245 (61.0)	109 (55.1)
mild	177 (29.5)	118 (29.4)	59 (29.8)
moderate	52 (8.7)	27 (6.7)	25 (12.6)
moderately severe	10 (1.7)	7 (1.7)	3 (1.5)
severe	2 (0.3)	2 (0.5)	0 (0.0)
NA	5 (0.8)	3 (0.7)	2 (1.0)
GAD-7 (degree of anxiety symptom severity) ^d)^, n (%)			
no/minimal	396 (66.0)	272 (67.6)	124 (62.6)
mild	143 (23.8)	94 (23.4)	49 (24.7)
moderate	17 (2.8)	6 (1.5)	11 (5.6)
severe	40 (6.7)	26 (6.5)	14 (7.1)
NA	4 (0.7)	4 (1.0)	0 (0.0)

^a)^Multiple-choice answer options in the domains: i) acquiring knowledge ii) working carefully iii) regular recovery/rest periods, iv) regular vacations, v) regular physical activity, vi) relaxation exercises, vii) talking to people of trust, viii) No active or conscious mental health protection. The sum of total mental health protection strategies exceeds the overall number of participants due to multiple choice answer options. Ambiguous answers (i.e., choice of ≥1 mental health protection strategies and ‘no protection strategy’) were categorized as no protection strategy (n = 58). The final variable was coded as ‘none’ (“I do not actively protect my mental health”), and ‘any’ (having ticked at least on protection strategy). Percentages are based on total participants (n = 600)

^b)^Sum of total physical and mental health issues exceeds overall number of participants with a self-reported health issue due to multiple choice answer options. Percentages are based on total participants (n = 600)

^c)^Sample sizes for the depression models without missing values in the outcome (PHQ-9), explanatory, or confounding variables: N_total_ = 554, N_men_ = 377, N_women_ = 177

^d)^ Sample sizes for the anxiety models without missing values in the outcome (GAD-7), explanatory, or confounding variables: N_total_ = 555, N_men_ = 376, N_women_ = 179

### Prevalence of depression and anxiety symptoms in the FarmCoSwiss cohort compared to the Swiss Health Survey

**Fig. 2:**
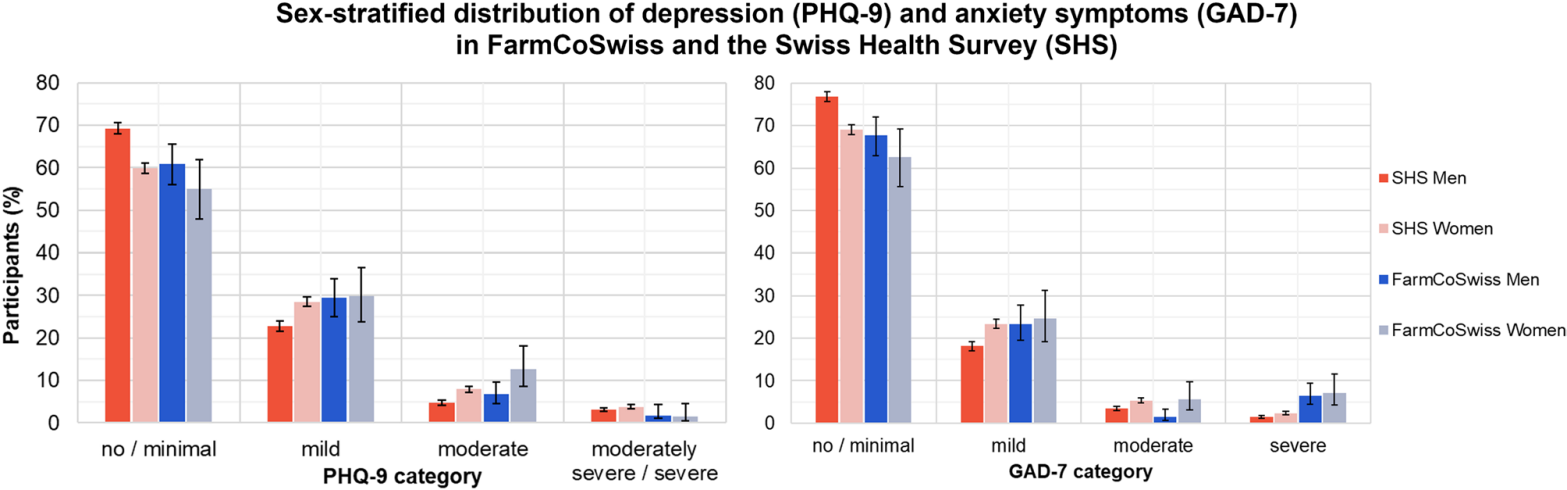
Sex-stratified frequency distribution of the PHQ-9 (depression symptoms) and GAD-7 (anxiety symptoms) categories in the *FarmCoSwiss* cohort at follow-up (*n* = 600, men = 402, women = 198; PHQ-9: missing = 5; GAD-7: missing = 4) and in the Swiss Health Survey (SHS) of 2022 (PHQ-9: *n* = 18,447, men = 8,547, women = 9,900; GAD-7: *n* = 18,688, men = 8,627, women = 10,061). Error bars indicate the 95% confidence interval (CI).

Figure 2 displays the percentage of *FarmCoSwiss* and SHS 2022 participants in each PHQ-9 and GAD-7 category, stratified by sex. Unstratified results can be found in the appendix (Figure A1). Comparisons are descriptive and no statistical tests were conducted.

About half of *FarmCoSwiss* participants reported no depression and anxiety symptoms (*n* = 314, 52.3%). The frequency of mild and moderate depression symptoms in the *FarmCoSwiss* cohort was 29.5% and 8.7%, respectively. In the SHS, these frequencies amounted to 25.7% for mild and 6.4% for moderate depression symptoms. Moderately severe or severe depression symptoms were reported by 3.4% of SHS and 2.0% of *FarmCoSwiss* participants. Regarding anxiety, the majority of both samples declared no or minimal symptoms, with a frequency of 66.0% in the *FarmCoSwiss* cohort and 72.9% in the SHS. Mild anxiety symptoms were prevalent in 20.8% of SHS and 23.8% of *FarmCoSwiss* participants. A total of 4.4% of SHS and 2.8% of *FarmCoSwiss* participants reported moderate anxiety symptoms, while severe anxiety symptoms were prevalent in 1.9% of the SHS and 6.7% of the *FarmCoSwiss* sample.

Regarding sex differences, men reported symptoms of depression and anxiety less frequently than women in both study samples. Women generally reported more mild and moderate depression and anxiety symptoms than men.

### Factors associated with depression and anxiety symptoms

**Fig. 3:**
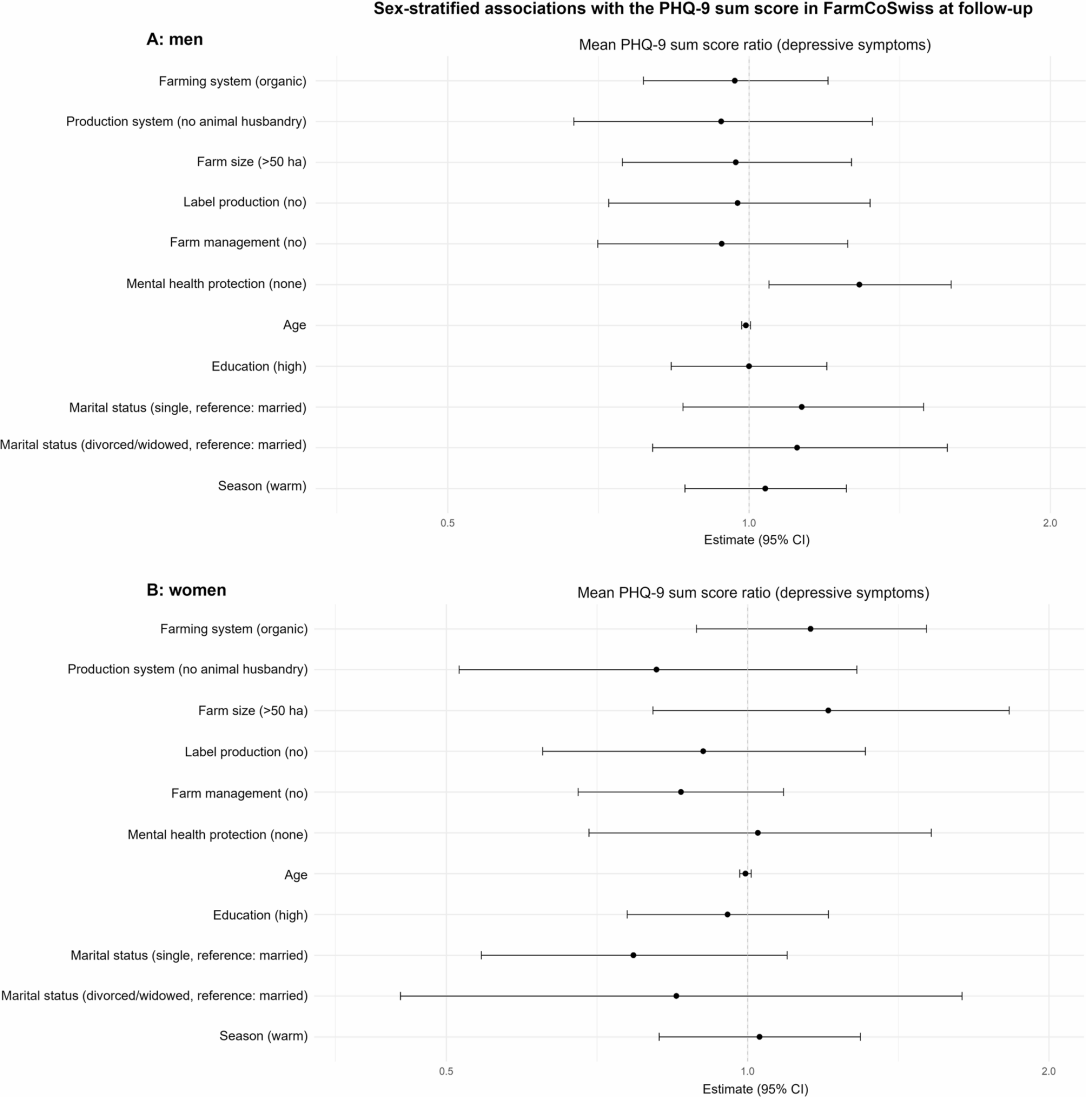
Forest plot of the results of the sex-stratified negative binomial regression model (log link), showing the association between farming system (non-organic, organic), production system (animal husbandry/mixed, no animal husbandry), farm size [hectares] (≤ 50, > 50), label production (yes, no), farm management position (yes, no), and individual-based mental health protection strategies (any, none) with the PHQ-9 sum score (depression symptoms). Panel A shows the results for men (*n* = 377), and panel B for women (*n* = 177). Effect estimates are back-transformed (exponentiated). Effect estimates indicate a lower/higher level of depression symptoms for the category indicated in parenthesis compared to the reference category. Estimates of categorical variables represent the ratio of mean PHQ-9 sum scores between the respective variable’s level (indicated in parentheses) and the reference category. Continuous variables represent the PHQ-9 sum score ratio associated with a one-unit increase in the respective variable. Bars indicate the lower and upper end of the 95% confidence interval. The model was adjusted for all variables, including age, education, marital status, and season in which individuals participated in the follow-up questionnaire

Figure 3 shows the negative binomial regression results of the mutually adjusted model exploring the cross-sectional association of the PHQ-9 sum score with farm-related characteristics and with the binary mental health protection strategy variable. Detailed regression results (estimates, *p*-values, and 95% confidence intervals (CIs) can be found in the appendix (Table A2). None of the farm variables were associated with depression symptoms in either men or women. In men only, implementing any of the listed mental health protection strategies was associated with a 22.4% lower PHQ-9 sum score (back-transformed estimate = 1.289, 95% CI = [1.047, 1.592]) as compared to those who did not actively use any mental health protection strategy. Negative binomial regression results of the mutually adjusted model investigating the cross-sectional association of the GAD-7 sum score with farm-related characteristics and the binary mental health protection strategy variable revealed results highly similar to those of the PHQ-9 model (Table A3). There was no association between farm variables and anxiety symptoms. Actively protecting one’s mental health was associated with a 31.5% lower GAD-7 sum score exclusively in men (1.460, [1.163, 1.840]).

Sensitivity analysis excluding participants with ambiguous answers in the mental health protection strategy question (*n* = 58) revealed no considerable changes in the above reported associations of farm-related characteristics and mental health protection with the PHQ-9 and GAD-7 sum scores (for detailed model outputs, see appendix Tables A4 and A5). The negative association between mental health protection strategies and the PHQ-9 and GAD-7 sum scores became stronger in both men (PHQ-9: 1.483, [1.105, 1.318]; GAD-7: 1.787, [1.301, 2.491]) and women (PHQ-9: 1.366, [0.867, 2.196]; GAD-7: 1.545, [0.968, 2.518]). While the effects became more similar in size and direction between men and women, they remained statistically significant only in men.

## Discussion

### Mental health in male and female farmers

We conducted descriptive analysis of the *FarmCoSwiss* and SHS data on depression and anxiety symptom prevalence. While comparisons between two distinct study populations should be interpreted with caution, our findings indicate that at least mild depression and anxiety symptoms in the farmers’ cohort might be more prevalent (PHQ-9: 41.6%, GAD-7: 33.3%) than in the Swiss general population (PHQ-9: 34.1%, GAD-7: 27.1%) for both men and women. About half of *FarmCoSwiss* participants reported no symptoms of either depression or anxiety. Men generally reported lower symptom prevalence than women in both samples.

While the PHQ-9 and GAD-7 instruments serve as screening tools that cannot be used for clinical diagnoses, the suggested finding of poorer mental health in farming communities compared to the general population corresponds to agricultural health research worldwide and in Switzerland [51]. In Switzerland, a longitudinal investigation of national data revealed a higher suicide rate in male farmers compared to non-farming men in the general population [34]. In a cross-sectional study published in 2017, farmers reported a higher burnout prevalence than the general population in Switzerland [56]. Compared to international research investigating depression and anxiety symptoms in farming populations using the PHQ-9 and GAD-7 instruments, the percentage of *FarmCoSwiss* participants reporting at least mild depression or anxiety symptoms was lower than in two farming samples from the US, but higher than in a study conducted in India [57–59]. However, these study samples are not directly comparable to the *FarmCoSwiss* sample in terms of age- and sex-distribution. While male and young farmers make up the majority of the farming samples in these studies, moderate depression and anxiety symptoms measured with the PHQ-9 and GAD-7 were also found to be more prevalent in a sample of female farmers in Ecuador as compared to female *FarmCoSwiss* participants [60]. Studies conducted in the US, Australia, UK, and Norway suggest overall poorer mental health in farmers as compared to the non-farming population or non-farming manual workers [4, 5, 61–64]. Yet, global evidence remains inconsistent overall, as other findings from Norway, the UK, or China, and thus in some cases from the same country, suggest no differences in mental health outcomes between the general population and farming populations, or in some cases even a higher prevalence in the general population [12, 20, 65]. However, we recently investigated human flourishing, i.e., the ability to thrive, in the *FarmCoSwiss* cohort and results suggest a similar pattern of lower flourishing in the farming population as compared to an urban sample from the Swiss general population [39].

We found depression and anxiety symptom prevalences in male and female *FarmCoSwiss* participants comparable to sex-specific patterns observed in the Swiss general population. Women generally reported more, and in particular more moderate, depression and anxiety symptoms. Female *FarmCoSwiss* participants also more often disclosed to have had a mental health issue in the past 12 months as compared to men. Data from other high-income countries, such as Canada, France, Japan, and the US, indicate that female farmers have higher depression prevalence or severity [28, 66–68]. In the UK, a study from 2023 reported mild anxiety symptoms in a third of female farmers, and at least moderate anxiety symptoms in about a quarter of participating women [27]. In comparison, anxiety symptom prevalences in both male and female *FarmCoSwiss* participants were much lower (mild: 24%, at least moderate: 10%). Although there is a body of global epidemiological evidence that aligns with our findings of women in farming communities generally reporting poorer mental health, research about women’s mental health in agriculture remains comparatively scarce [37]. Yet, a similar picture of poorer mental health in female farmers as compared to male farmers also presents in two recent publications within the *FarmCoSwiss* cohort using different mental health indices. Findings indicate lower flourishing values and lower mental health-related quality of life in female farmers as compared to their male colleagues [39, 40]. This suggests a robust finding in our study population measurable with different mental health-related instruments. It further aligns with findings from general population studies reporting higher depression and anxiety prevalences in women as compared to men [31, 69].

### Depression and anxiety symptoms in the context of farm characteristics and mental health protection strategies

In the present study, farm-related characteristics were not associated with depression or anxiety symptoms. The active implementation of mental health protection strategies were associated with lower depression and anxiety symptoms among men only.

#### Farm-related characteristics

Research exploring associated factors with farmers’ mental health frequently reports a complex set of different personal, economic, and societal challenges, environmental pressures, and farm-related characteristics [51]. However, evidence on the specific influence of farm-related characteristics on farmers’ mental health in high-income countries is inconclusive. Dairy farmers in Finland, Ireland, and the UK have been found to report poorer mental wellbeing than other livestock and arable farm types [21, 25, 70]. Studies from France suggest particularly high depression risk and suicide mortality rates for cattle and dairy farmers as compared to other production activities [71, 72]. In studies conducted in Japan and Norway, no association between the prevalence of depressive symptoms and animal husbandry was found [10, 24]. Often cited factors associated with farming, and livestock farming in particular, and poorer mental health include economic crises related to milk prices, food safety and quality regulations, time pressure and poor sleep, little time for holidays and leisure, and bovine disease outbreaks [8, 72–75]. However, a study of a representative sample of Swiss farmers investigating predictors of burnout did not find any association with farm-related characteristics, but identified bad financial situations, time pressure, work-family conflicts, and administration tasks as the main predictors, the latter only for women [33]. Similarly, we found psychosocial hazards, including interpersonal conflicts, sleep problems, stress, and loneliness to be negatively associated with human flourishing within the *FarmCoSwiss* cohort [39]. Thus, farm-related characteristics in our study might represent a proxy for other stressors, such as economic hardship or high workload. In general populations, the link between lower socio-economic status and poorer mental health is well established [76]. Given that farmers’ average wages in Switzerland are well below the median comparative income and average working hours exceed national maximum working hours, future research may investigate the association of farm incomes and other farm-related stressors with mental health symptoms [77]. Overall, the lack of an association between farm-related characteristics and mental health suggests that mental health challenges in the farming population may be driven by individual and contextual factors, such as social support or economic stressors. Future research may therefore explore the role of psychosocial resilience, community support, or financial stability to improve mental health support and prevention interventions in Swiss agricultural populations.

#### Mental health protection strategies

Regarding individual-based mental health protection strategies, we found that more women implement mental health protection strategies (87.9%) than men (76.1%). Regression analyses revealed 22.4% and 31.5% lower expected PHQ-9 and GAD-7 sum scores, respectively, in male farmers who implemented some mental health protection strategies as compared to those who stated they did not actively protect their mental health. This negative association between protection strategies and depression and anxiety symptoms was not found in women, although sensitivity analyses yielded larger effect estimates more similar to those in men. These findings may indicate that the individual-based mental health protection strategies inquired about in the follow-up survey, such as regular rest, physical activity, or the pursuit of a hobby, are more effective in protecting against farm-specific stressors for male participants than for female farmers. It is conceivable that advice and services available for protecting Swiss farming communities’ mental health are predominantly targeted to male farmers and ignore specific needs of female farmers. Women in agriculture potentially deal with multiple burdens, such as farming tasks, household chores, child and family care, and possible off-farm work, which could render the listed mental health protection strategies less effective. Qualitative research in the US, Canada, and Australia shows that male farmers implement similar mental health protection strategies as in the *FarmCoSwiss* cohort, including maintaining a work-life balance, physical activity, spending time with others, faith, or acquiring new knowledge on mental health [78–80]. Mental health strategies reported by farmers in other studies and assessed in the present study can be broadly categorized into different topics, such as detachment from work (breaks, hobbies, mindfulness), mindset (gratefulness, faith, humor), and interaction with others (talking to family, colleagues, professionals) [78, 80]. However, male farmers in particular have also reported to engage in negative strategies, such as social isolation and substance abuse, which were not assessed in the present study [79, 80]. Global research in psychology consistently shows that women are more frequently affected by internalizing disorders such as depression or anxiety, while men are more often diagnosed with externalizing disorders, including substance abuse or antisocial personality disorder [31, 81]. Thus, our finding that mental health protection strategies were associated with better mental health only in men may also be due to reverse causality. Specifically, the lower baseline level of depression and anxiety symptoms in our sample, as well as sex differences in the types of protection strategies employed, may offer alternative explanations. Future research may therefore be interested in investigating negative mental health protection strategies and potential differences in mental health protection strategies of male and female farmers in Switzerland. It may also be of interest to assess other mental health outcomes, including those more commonly associated with externalizing disorders, such as substance use. While self-management strategies cannot replace professional support in case of mental health struggles, more evidence on positive and negative mental health strategies and their longitudinal impact on mental health in the Swiss agricultural context may better inform future mental health interventions.

#### Mental health support seeking

Women who reported a mental health issue in the past 12 months more often sought help (42.4%) than men (30.2%), although treatment sought for mental health issues was drastically lower (35.5%) as compared to treatment seeking for physical health issues (81.5%), in both men and women. Notably, these sex-specific findings may indicate that women in the *FarmCoSwiss* study might have poorer mental health, but also higher mental health service usage and individual-based mental health protection. Research on health care utilization among farming communities has shown that stereo-typical gender views and traditional values may affect health care seeking behavior in men and women [78, 82, 83]. Stigma associated with mental health problems and values such as pride, self-reliance, or a lack of knowledge have been identified as key barriers mainly in male farmers to seek mental health support [12, 82, 83]. General population studies in Western countries further confirm that men are less willing to seek mental health support and are less likely to receive treatment, even when controlling for prevalence rates [84]. Explanations for this sex difference relate to gender role socialization and traditional masculinity norms, promoting values such as the perception of negative emotions as a sign of weakness. Furthermore, rural communities in general have been found to prefer informal support and self-reliance over professional help [85]. However, research has also identified generational and individual differences between ideals and values present in farmers, such as the perception of asking for aid as a sign of weakness and the admiration for men who have the courage to seek help [82]. For women, a qualitative study in Ireland identified the scarcity of women-specific support services and mental health stigma as main barriers to seek support [29]. In Switzerland, mental ill-health in farming communities has gained attention in recent years. Major agricultural associations offer information on mental health and agricultural-specific mental health services on a national and regional level are available [86]. Nonetheless, the availability of mental health care services in rural areas in Switzerland remains challenging [36]. In general, community-led approaches, role models, and digital interventions have been shown to be promising approaches to improve mental health awareness and service utilization in farming communities in Australia and Ireland [85, 87, 88]. The present study’s results highlight potential benefits of sex-specific approaches and further research on mental health support needs in farming communities in Switzerland.

### Strengths and limitations

Evidence on farmers’ physical and mental health in Switzerland is scarce. *FarmCoSwiss* as the first national agricultural cohort study can therefore aid in the identification and long-term observation of pressing health issues and in contributing to agricultural and public health research in Switzerland. Observing and examining farmers’ mental health over time remains crucial in view of present and future environmental and societal challenges affecting agriculture. The present study offers insights into farmers’ mental health in Switzerland and sex-specific differences, allowing for the future temporal investigation of mental health outcomes and associated risk and protective factors.

However, some study limitations need to be acknowledged. First, our study sample may not be representative of the Swiss farming population. In addition to the healthy worker effect, selection bias cannot be excluded. Thus, our sample may overrepresent individuals who are more motivated to participate in research or are particularly health-conscious, potentially resulting in a cohort with better overall mental and physical health than the Swiss farming population. Moreover, no national registry data for farmers were available to allow for population-based sampling at baseline. Yet, we attempted to increase representativeness by promoting our study through different agricultural networks, such as fruit production, livestock and organic farming organizations, and associations for female farmers. Specifically, our study sample at follow-up comprised more organic farms (Switzerland: 16%, *FarmCoSwiss*: 23%), more farms with livestock (Switzerland: 67%, our *FarmCoSwiss*: 91%), and more farms with a size of ≥ 50 ha (Switzerland: 7%, *FarmCoSwiss*: 12%) than the Swiss national farming population [89]. In addition, we achieved a high participation rate at follow-up for this current study. Although attrition bias between baseline and the first follow-up may not be ruled out, we did not observe major differences between population and farm-related characteristics among farmers who participated in the follow-up questionnaire and those who did not, except for a slightly higher percentage of highly educated individuals at follow-up (baseline: 41%, follow-up: 49%). The representativeness of cohort participants at baseline is discussed in detail in a previous publication [40].

Second, missing values were minimized by requiring participants to respond to all questionnaire items in the online questionnaire. Yet, individuals answering the questionnaire on paper or abandoning the survey resulted in missing values. Since missing values made up less than 3% for all variables of interest, we refrained from using imputation methods and introduction of bias is unlikely.

Third, descriptive analyses comparing the prevalence of depression and anxiety symptoms in the *FarmCoSwiss* cohort and in the SHS (Swiss general population) require cautious interpretation, as the SHS also includes adolescents aged 15 to 18 years and unemployed individuals. Additionally, PHQ-9 and GAD-7 data from the SHS was available from 2022, while F*armCoSwiss* data was collected in 2024. Thus, short-term COVID-19 pandemic-related effects in the SHS sample cannot be ruled out. However, we would expect the true prevalence difference between farmers and the general population to be even greater after considering a potential COVID-19 pandemic impact, which likely had a stronger effect on the 2022 SHS data than on the 2024 *FarmCoSwiss* data. In addition to the above-mentioned selection bias, these differences in study population and timing suggest that the observed difference in the present descriptive analysis may be an underestimation. Moreover, as the SHS and *FarmCoSwiss* samples were drawn within different studies and from different populations, we refrained from calculating risk estimates. As Switzerland lacks a national (occupational) cohort, the Swiss research environment may prioritize the establishment of such a cohort to provide evidence on mental health risk estimates for farmers and non-farming workers.

Fourth, the PHQ-9 and GAD-7 instruments were designed to screen for depression and anxiety symptoms and are meant for provisional diagnosis, particularly in primary health care settings [44, 47]. Specificity and sensitivity of both tools were found to be around 85% for a sum score cut-off at ≥ 10 (PHQ-9: sensitivity = 88%, specificity = 85%; GAD-7: sensitivity = 89%, specificity = 82%) [47, 90]. Hence, false positive findings may be of concern. Future validation studies of these instruments in occupational settings might aid in a more accurate interpretation of the present results. Furthermore, the longitudinal design of the cohort will facilitate investigation of the longitudinal consequences of reported symptoms on the incidence of mental health diagnoses.

Fifth, the measure of individual mental health protection strategies was informed by previous literature but was not based on a formally validated instrument. While the strategies listed include widely recognized mental health protecting behaviors, the lack of a validated instrument may limit comparability across studies and introduce potential measurement bias. Future studies may investigate the validity and reliability of this measure in other agricultural settings.

Sixth, despite the longitudinal design of *FarmCoSwiss* the current analysis is entirely cross-sectional and based on self-report data. Reverse causation and social desirability bias may therefore be of concern, particularly regarding the direction of association between reported mental health protection strategies and the level of mental health symptoms. Furthermore, we do not know whether participants were undergoing treatment for a mental health issue at the time of the survey. Such treatment might have influenced their ability to implement certain mental health protection strategies or reduced their depression or anxiety symptoms. However, only 13% of participants reported having experienced a mental health issue in the past 12 months, and among those, just 36% (*n* = 27) reported having sought treatment. Hence, the number of individuals under treatment at the time of the survey is probably small. In addition, the presumably long-term implementation of mental health protection strategies calls for a depression and anxiety symptom assessment covering a longer time period than two weeks before questionnaire completion. However, despite potential reverse causality issues, the observed association was present only among men, which warrants further research into gender-specific associations or reporting patterns. Of note, sample size for female farmers reporting no mental health protection strategies was substantially smaller (9.4%) than for male farmers (21.4%). Thus, limited statistical power may explain the lack of an association of mental health protection strategies with depression and anxiety symptoms in women.

Furthermore, more vulnerable groups are currently underrepresented in our cohort, including adolescents or migrant and employed farm workers [91]. In contrast, women represent one third of participants at baseline and at follow-up, corresponding to roughly one third of women in the Swiss agricultural workforce [92]. Lastly, the self-reported measure of sex does not capture the full spectrum of gender identities or roles and may limit the generalizability of findings related to sex/gender differences. Future follow-up rounds may assess gender (roles) and expand the scope to include children or young adults and seasonal workers.

## Conclusion

The present study provides evidence on farmers’ mental health in Switzerland. Our findings indicate potentially poorer mental health in the Swiss farming community, and in particular in female farmers, as compared to the general population, but underlaying reasons remain unclear. There was no association of farm-related variables, such as production system, management position, or label production with depression or anxiety symptom severity. Individual-based mental health protection strategies were negatively associated with depression and anxiety symptoms in men only. As the majority of participants reported mental health protection strategies, future longitudinal studies may evaluate which mental health protection strategies may be most effective and how they can be promoted for both men and women. The *FarmCoSwiss* cohort offers the opportunity to investigate the sex-specific needs and longitudinal development of physical and mental health outcomes in the Swiss agricultural workforce in-depth. It can thereby contribute to tailored mental health information campaigns and interventions in the Swiss cultural and agricultural context.

## Supplementary Information

Supplementary Material 1.

## Data Availability

The datasets generated and/or analyzed during the current study are not publicly available due to reasons of sensitivity and are available from the corresponding author upon reasonable request, and under a data use agreement only. Data are located in controlled access data storage at the Swiss Tropical- and Public-Health Institute.

## Appendix

**Table 3:** FarmCoSwiss participant and farm-related characteristics from the baseline survey (no follow-up data is presented here), stratified for individuals who later did or did not participate in the first follow-up questionnaire

	Baseline total	Baseline: Follow-up 1 non-participants	Baseline: Follow-up 1 participants ^a)^
Sociodemographic variables			
Participants, n (%)	878 (100)	267 (30.4)	600 (68.3)
Sex, n (%)			
female	300 (34.2)	96 (36.0)	198 (33.0)
male	578 (65.8)	171 (64.0)	402 (67.0)
Age [years], mean (median, min-max)			
mean (± SD)	48.8 (± 11.4)	48.3 (± 11.5)	48.9 (± 11.3)
min-max	21–86	21–86	24–82
missing	3 (0.3)	1 (0.4)	2 (0.3)
Highest educational level ^b)^, n (%)			
low/middle	460 (52.4)	153 (57.3)	301 (50.2)
high	411 (46.8)	110 (41.2)	296 (49.3)
missing	7 (0.8)	4 (1.5)	3 (0.5)
Farm-related characteristics			
Farming system, n (%)			
non-organic	678 (77.2)	209 (78.3)	462 (77.0)
organic	195 (22.2)	56 (21.0)	136 (22.7)
missing	5 (0.6)	2 (0.7)	2 (0.3)
Production system, n (%)			
animal husbandry ^c)^	785 (89.4)	228 (85.4)	548 (91.3)
no animal husbandry	82 (9.3)	34 (12.7)	48 (8.0)
missing	11 (1.3)	5 (1.9)	4 (0.7)
Farm size [hectares], n (%)			
≤ 50	774 (88.2)	236 (88.4)	528 (88.0)
> 50	101 (11.5)	30 (11.2)	71 (11.8)
missing	3 (0.3)	1 (0.4)	1 (0.2)
Farm management position, n (%)			
yes	655 (74.6)	201 (75.3)	450 (75.0)
no	211 (24.0)	62 (23.2)	144 (24.0)
missing	12 (1.4)	4 (1.5)	6 (1.0)

^a)^ Excluding individuals who participated in the follow-up questionnaire but were excluded in the present analyses (n = 11)

^b)^Education: low = mandatory primary and secondary education (≤9 years), middle = vocational training or high school (≤12 years), high = higher technical or vocational school or university (>12 years)

^c)^Includes any farm with animal husbandry, either with or without crop production

**Table 4:** Negative binomial regression analysis results (log scale, back transformed) investigating the association of farm variables and health protection strategies with the PHQ-9 sum score (depression symptoms), stratified by sex. The model was adjusted for age, education, marital status, and season. P-values below 0.05 are marked in bold.

Variable	Estimate (sum score ratio)	*p*-value	95% CI ^b)^
**Men** (*n* = 377) ^a)^			
Intercept	5.835	0.000	3.419, 10.011
Farming system ^c)^	0.968	0.764	0.784, 1.199
Production system ^d)^	0.938	0.717	0.668, 1.328
Farm size (> 50 ha)^e)^	0.970	0.819	0.747, 1.266
Label production ^f)^	0.974	0.868	0.724, 1.321
Management ^g)^	0.939	0.664	0.706, 1.255
Mental health protection ^h)^	1.289	**0.017**	**1.047**,** 1.592**
Age	0.993	0.133	0.983, 1.003
Education (high)	1.000	0.999	0.836, 1.196
Marital status (single)	1.129	0.384	0.859, 1.494
Marital status (divorced/widowed)	1.117	0.522	0.801, 1.578
Season (warm)	1.038	0.688	0.863, 1.251
**Women** (*n* = 177) ^**a)**^			
Intercept	6.985	0.000	3.573, 13.704
Farming system ^c)^	1.156	0.282	0.889, 1.509
Production system ^d)^	0.811	0.362	0.515, 1.286
Farm size (> 50 ha) ^e)^	1.204	0.363	0.804, 1.825
Label production ^f)^	0.903	0.598	0.624, 1.311
Management ^g)^	0.858	0.194	0.677, 1.086
Mental health protection ^h)^	1.024	0.907	0.694, 1.526
Age	0.995	0.412	0.982, 1.008
Education (high)	0.955	0.695	0.758, 1.204
Marital status (single)	0.769	0.139	0.542, 1.095
Marital status (divorced/widowed)	0.849	0.613	0.450, 1.638
Season (warm)	1.028	0.814	0.816, 1.296

^a)^ Sample sizes without missing values in any of the outcome, explanatory, or confounding variables

^b)^95% CI: 95% confidence interval

^c)^organic, reference category non-organic

^d)^no animal husbandry/mixed, reference category animal husbandry

^e)^[hectares], reference category <50 ha

^f)^no label production, reference category label production

^g)^no (co-)management position on farm, reference category (co-)management position

^h)^None of the seven mental health protection strategies implemented, reference category any active mental health protection

**Table 5:** Negative binomial regression analysis results (log scale, back transformed) investigating the association of farm variables and health protection strategies with the GAD-7 sum score (anxiety symptoms), stratified by sex. The model was adjusted for age, education, marital status, and season. P-values below 0.05 are marked in bold.

Variable	Estimate (sum score ratio)	*p*-value	95% CI ^b)^
**Men** (*n* = 376) ^a)^			
Intercept	5.831	0.000	3.237, 10.572
Farming system ^c)^	1.001	0.991	0.795, 1.266
Production system ^d)^	1.020	0.920	0.706, 1.492
Farm size (> 50 ha) ^e)^	1.189	0.231	0.899, 1.586
Label production ^f)^	1.055	0.754	0.763, 1.475
Management ^g)^	1.038	0.816	0.758, 1.430
Mental health protection ^h)^	1.460	**0.001**	**1.163**,** 1.840**
Age	0.986	**0.011**	**0.976**,** 0.997**
Education (high)	1.047	0.649	0.860, 1.274
Marital status (single)	1.070	0.659	0.792, 1.458
Marital status (divorced/widowed)	0.989	0.954	0.679, 1.460
Season (warm)	1.173	0.123	0.956, 1.441
**Women** (*n* = 179) ^**a)**^			
Intercept	8.405	0.000	4.165, 17.089
Farming system ^c)^	1.174	0.265	0.887, 1.558
Production system ^d)^	1.084	0.737	0.676, 1.757
Farm size (> 50 ha) ^e)^	1.136	0.562	0.738, 1.769
Label production ^f)^	0.701	0.099	0.465, 1.056
Management ^g)^	0.900	0.402	0.699, 1.158
Mental health protection ^h)^	1.160	0.505	0.769, 1.767
Age	0.987	0.063	0.974, 1.001
Education (high)	0.943	0.636	0.738, 1.204
Marital status (single)	0.532	**0.001**	**0.360**,** 0.785**
Marital status (divorced/widowed)	0.802	0.523	0.407, 1.615
Season (warm)	1.053	0.680	0.823, 1.349

^a)^ Sample sizes without missing values in any of the outcome, explanatory, or confounding variables

^b)^95% CI: 95% confidence interval

^c)^organic, reference category non-organic

^d)^no animal husbandry/mixed, reference category animal husbandry

^e)^[hectares], reference category <50 ha

^f)^no label production, reference category label production

^g)^no (co-)management position on farm, reference category (co-)management position

^h)^None of the seven mental health protection strategies implemented, reference category any active mental health protection

**Table 6:** Sensitivity analysis excluding participants with ambiguous answers in the multiple-choice question on mental health protection strategies (n = 58). Negative binomial regression analysis results (log scale, back-transformed) investigating the association of farm variables and health protection strategies with the PHQ-9 sum score (depression symptoms), stratified by sex. The model was adjusted for age, education, marital status, and season. P-values below 0.05 are marked in bold.

Variable	Estimate (sum score ratio)			*p*-value	95% CI ^b)^
**Men** (*n* = 326) ^a)^					
Intercept	6.096			0.000	3.448, 10.843
Farming system ^c)^	0.921			0.488	0.732, 1.163
Production system ^d)^	0.974			0.883	0.687, 1.394
Farm size (> 50 ha) ^e)^	0.866			0.324	0.651, 1.158
Label production ^f)^	1.046			0.782	0.768, 1.437
Management ^g)^	0.973			0.858	0.722, 1.318
Mental health protection ^h)^	1.483			**0.010**	**1.105**,** 2.012**
Age	0.992			0.126	0.981, 1.003
Education (high)	0.968			0.735	0.799, 1.172
Marital status (single)	1.165			0.320	0.864, 1.582
Marital status (divorced/widowed)			1.129	0.506	0.795, 1.625
Season (warm)	1.072			0.490	0.878, 1.311
**Women** (*n* = 170) ^**a)**^					
Intercept	6.032			0.000	3.075, 11.862
Farming system ^c)^	1.144			0.319	0.878, 1.494
Production system ^d)^	0.748			0.206	0.474, 1.186
Farm size (> 50 ha) ^e)^	1.238			0.309	0.818, 1.898
Label production ^f)^	0.943			0.770	0.641, 1.396
Management ^g)^	0.882			0.286	0.698, 1.116
Mental health protection ^h)^	1.366			0.192	0.867, 2.196
Age	0.997			0.681	0.984, 1.010
Education (high)	0.963			0.745	0.763, 1.214
Marital status (single)	0.817			0.264	0.570, 1.174
Marital status (divorced/widowed)		0.838		0.578	0.448, 1.598
Season (warm)	1.022			0.851	0.812, 1.288

^a)^ Sample sizes without missing values in any of the outcome, explanatory, or confounding variables

^b)^95% CI: 95% confidence interval

^c)^organic, reference category non-organic

^d)^no animal husbandry/mixed, reference category animal husbandry

^e)^[hectares], reference category <50 ha

^f)^no label production, reference category label production

^g)^no (co-)management position on farm, reference category (co-)management position

^h)^None of the seven mental health protection strategies implemented, reference category any active mental health protection

**Table 7:** Sensitivity analysis excluding participants with ambiguous answers in the multiple-choice question on mental health protection strategies (n = 58). Negative binomial regression analysis results (log scale, back-transformed) investigating the association of farm variables and health protection strategies with the GAD-7 sum score (anxiety symptoms), stratified by sex. The model was adjusted for age, education, marital status, and season. P-values below 0.05 are marked in bold.

Variable	Estimate (sum score ratio)		*p*-value	95% CI ^b)^
**Men** (*n* = 325) ^a)^				
Intercept	6.475		0.000	3.458, 12.211
Farming system ^c)^	0.964		0.775	0.749, 1.244
Production system ^d)^	1.060		0.770	0.727, 1.566
Farm size (> 50 ha) ^e)^	1.119		0.476	0.826, 1.530
Label production ^f)^	1.149		0.436	0.821, 1.627
Management ^g)^	1.067		0.695	0.768, 1.493
Mental health protection ^h)^	1.787		**0.000**	**1.301**,** 2.491**
Age	0.985		**0.007**	**0.973**,** 0.996**
Education (high)	1.001		0.990	0.811, 1.236
Marital status (single)	1.073		0.677	0.770, 1.507
Marital status (divorced/widowed)		0.978	0.916	0.659, 1.475
Season (warm)	1.216		0.078	0.976, 1.518
**Women** (*n* = 172) ^**a)**^				
Intercept	7.299		0.000	3.644, 14.717
Farming system ^c)^	1.151		0.320	0.873, 1.521
Production system ^d)^	0.991		0.969	0.620, 1.594
Farm size (> 50 ha) ^e)^	1.146		0.539	0.741, 1.794
Label production ^f)^	0.764		0.214	0.501, 1.165
Management ^g)^	0.935		0.585	0.731, 1.197
Mental health protection ^h)^	1.545		0.079	0.968, 2.518
Age	0.990		0.130	0.976, 1.003
Education (high)	0.943		0.630	0.741, 1.199
Marital status (single)	0.573		**0.005**	**0.385**,** 0.851**
Marital status (divorced/widowed)		0.770	0.438	0.397, 1.515
Season (warm)	1.049		0.698	0.823, 1.337

^a)^ Sample sizes without missing values in any of the outcome, explanatory, or confounding variables

^b)^95% CI: 95% confidence interval

^c)^organic, reference category non-organic

^d)^no animal husbandry/mixed, reference category animal husbandry

^e)^[hectares], reference category <50 ha

^f)^no label production, reference category label production

^g)^no (co-)management position on farm, reference category (co-)management position

^h)^None of the seven mental health protection strategies implemented, reference category any active mental health protection
