# Supplementary material for: Depression and anxiety symptoms in male and female farmers: association with farm characteristics and mental health protection strategies in the FarmCoSwiss cohort
Source: BMC Public Health. 2025 Dec 4;26:101. doi: 10.1186/s12889-025-25407-z (PMC12781764; doi:10.1186/s12889-025-25407-z)
Supplement: Supplementary file 1 — Supplementary Material 1. [file 12889_2025_25407_MOESM1_ESM.docx]

**Depression and anxiety symptoms in male and female farmers: association with farm characteristics and mental health protection strategies in the FarmCoSwiss cohort**

**Authors**

P. Ammann^1,2^, A. Jeong^1,2^, G. Lovison^1,2^, J. Doetzer^1,2^, S. Fuhrimann^1,2^, M. Imboden^1,2^, K. Ingold^3,4,5^, M.S. Winkler^1,2^, N. Probst-Hensch^1,2,^*

^1^ Swiss Tropical and Public Health Institute, Allschwil, Switzerland

^2^ University of Basel, Basel, Switzerland

^3^ Department of Environmental Social Sciences, Eawag, Swiss Federal Institute of Aquatic Science and Technology, Überlandstrasse 133, 8600, Dübendorf, Switzerland

^4^ Institute of Political Science, University of Bern, Fabrikstrasse 8, 3012, Bern, Switzerland

^5^ Oeschger Centre for Climate Change Research, University of Bern, Hochschulstrasse 4, 3012, Bern, Switzerland

*** Corresponding author**

Prof. Dr. N. Probst-Hensch: nicole.probst@swisstph.ch

**Supplement**

**Contents**

[S1 Baseline Questionnaire in English (not used in data collection) 3](#_Toc211770466)

[S2 First Follow-up Questionnaire in English (not used in data collection) 20](#_Toc211770467)

## S1 Baseline Questionnaire in English (not used in data collection)

***NOTE: The questionnaire was originally administered in German, French, and Italian. An English translation in a simple layout is provided here for the purpose of transparency and review only. The English version was not used in data collection.***

**FarmCoSwiss: Questionnaire on the Health of Individuals and Their Partners in Swiss Agriculture**

Dear participant,

The Swiss Tropical and Public Health Institute (Swiss TPH) is conducting this questionnaire to assess the general health and well-being of individuals working in Swiss agriculture and their spouses or life partners (hereafter referred to simply as “partner”). Your participation will make an important contribution to evaluating and improving the health of the agricultural population in Switzerland. Please complete the questionnaire without interruption. It will take approximately 40 minutes to complete.

We take data protection and confidentiality very seriously. All information collected will be treated with **strict confidentiality**, analyzed in **encrypted** form, and used exclusively for scientific purposes.

Depending on whether you

a) work in agriculture yourself, or

b) work outside of agriculture but your partner works in agriculture,

you will answer slightly different questions throughout the questionnaire. Questions that can be skipped by certain participants are clearly marked with an arrow.

If you have any questions or need assistance, please contact the study team at 061 284 89 29 or farmcoswiss@swisstph.ch

Thank you very much for your participation!

Prof. Dr. Nicole Probst-Hensch

**Paper Questionnaire**

Please complete the questionnaire using a black or blue ballpoint pen. When finished, kindly return the completed questionnaire in the enclosed pre-addressed and prepaid envelope to the Swiss Tropical and Public Health Institute.

Thank you very much!

**Introductory Questions**

**0) Please indicate the date on which you are completing this questionnaire.**

___/___/_____ (DD-MM-YYYY)

**0.1) Do you work on a farm?**

*By "work," we mean individuals who are self-employed, employed (full-time or part-time), in training, doing seasonal work, or volunteering on a farm (e.g., as family members). Activities on the farm may include agricultural as well as administrative tasks.*

If no: Please proceed directly to Question 0.2.

Yes/No

**0.1.1) If you work on a farm: Are there other people who work on the same farm as you who are also participating in this survey (excluding your spouse/partner)?**

Yes/No/I don’t know

If yes: How many people? ______

**0.2) Does your spouse/partner also participate in this survey?**

Yes/No/I don’t know/I don’t have a spouse/partner

**0.2.1) If your partner is also participating in the survey, please provide their full name and date of birth. We require this information to link you with your partner.**

If no, don’t know, or no partner: Please proceed directly to the “Contact Information” section.

First name ________________

Last name ________________

Date of birth ________________

**Some questions in this questionnaire refer to the farm where you work. Please answer these questions based on the farm where you have spent the most time working in the past 12 months. If you do not work in agriculture yourself, please answer these questions for the farm where your partner has spent the most time working in the past 12 months. All information will be treated with strict confidentiality.**

**Contact Information**

**1.1) Please provide your first and last name.**

First name: ___________________

Last name: ___________________

**1.2) Have your residential and/or farm address, as provided during registration, changed since then?**

Yes/No

If yes, new residential address: ____________________________________

From what date is this new residential address valid? ___/___/_____ (DD-MM-YYYY)

If yes, new farm address: ____________________________________

From what date is this new farm address valid? ___/___/_____ (DD-MM-YYYY)

**1.3) Have your phone number and/or email address, as provided during registration, changed since then?**

Yes/No

If yes, new phone number: ________________________

If yes, new email address: ________________________

**Education**

**2.1) What is your highest level of education?** Please select only one answer.

Compulsory primary and secondary school/Apprenticeship or vocational school or business school/High school/Higher technical or vocational school/University or college/Other, please specify: ___________________________

**2.2) What is your basic agricultural training?** Multiple possible answers.

No basic agricultural training/Agricultural practitioner (EBA)/Agriculturist/Poultry specialist/Vegetable gardener/Fruit specialist/Winemaker/Wine technologist/Other, please specify: ___________________________

**2.3) What is your highest level of advanced agricultural training?** Multiple possible answers.

No advanced agricultural training/Farm household manager/Agricultural vocational exam (farm manager school)/Advanced vocational exam/master exam (farm manager school level 2)/Advanced technical school: Agricultural business specialist/Advanced technical school: Agricultural technician/Advanced technical school: Viticulture technician/Other, please specify: ___________________________

**2.4) How many years have you worked on a farm?** Please select only one answer.

Never/1–5 years/6–10 years/11–20 years/21–30 years/More than 30 years

**2.5) How many years have you lived on a farm?** Please select only one answer.

Never/1–5 years/6–10 years/11–20 years/21–30 years/More than 30 years

**2.6) Did you grow up on a farm?** Please select only one answer.

Yes/No/Other, please specify: ___________________________

**Employment**

**3.1) What is your professional position on the farm?** Multiple possible answers.

If you do not work on a farm, please proceed directly to Question 3.1.1.

Farm operator or co-operator (farm management)/Working in the family farm (with income subject to social security contributions)/Working in the family farm (without income subject to social security contributions)/Employee with supervisory responsibilities (managing staff)/Employee without supervisory responsibilities (no staff management)/Retired but still working/In training/Other, please specify: ___________________________

**3.1.1) If you do not work on a farm:** **What is your current professional position?** Multiple possible answers.

If you do work on a farm, please proceed directly to Question 3.2.

Self-employed/Employee with supervisory responsibilities (managing staff)/Employee without supervisory responsibilities (no staff management)/Retired/In training/Homemaker (housewife/househusband)/Disabled or unable to work/Seeking employment/Other, please specify: ___________________________

**3.2) Do you work full-time or part-time on a farm?** Please select only one answer.

*Work is considered full-time if it clearly exceeds other gainful activities in terms of both economic importance and time commitment.*

If you do not work on a farm, please proceed directly to Question 3.2.1.

Full-time/Part-time/Other, please specify: ___________________________

**3.2.1) If you do not work on a farm: What is your employment workload in your current occupation?** Please select only one answer.

If you do work on a farm, please proceed directly to Question 3.4.

Full-time/Part-time/Other, please specify: ___________________________

**3.3) If you do not work on a farm: In which economic sector are you currently employed?** Multiple possible answers.

If you do work on a farm, please proceed directly to Question 3.4.

Manufacturing (e.g., production of goods) or Energy supply/Construction/Trade and repair services or Transportation and storage or Hospitality industry/Information and communication/Financial and insurance services/Real estate or other business services/Professional, administrative, technical, or scientific services/Public administration or Education or Health and social services/Arts or Entertainment or other service industries/Other, please specify: ___________________________

**3.4) Do you also work in an occupation outside of agriculture?** Please select only one answer.

If you do not work on a farm, please proceed directly to the section “General Health.”

No/Yes, year-round and less than 50% of working time/Yes, year-round and 50% or more of working time/Yes, only during the colder season and less than 50% of working time/Yes, only during the colder season and 50% or more of working time/Yes, only during the warmer season and less than 50% of working time/Yes, only during the warmer season and 50% or more of working time/Other, please specify: ___________________________

If yes: Please indicate the job title of your occupation outside of agriculture.

Job title: ___________________________

**Farm Management and Household Structure**

**If you do not work on a farm yourself, please skip all questions in the section “Farm Management and Household Structure” and proceed directly to the section “General Health.”**

**4.1) Who owns the farm?** Multiple possible answers.

You yourself/Your spouse or partner/Your father or mother/Your brother or sister/Another family member/Unrelated person/Other, please specify: ___________________________

**4.2) What is the farm’s primary production focus?** Multiple possible answers.

Livestock farming – dairy production/Livestock farming – meat production/Intensive farming – pig farming/Intensive farming – poultry farming/Arable (crop) farming/Specialized crop farming (e.g., fruit, vegetables, berries, or vineyards)/Horticulture/Forestry/Other, please specify: ___________________________

**4.3) Which crops are cultivated / managed on the farm, and approximately how many hectares are used for each crop?** Multiple possible answers.

*If no crops are grown on the farm, please skip this question.*

Grains <5ha 5–10 11–20 21–50 >50ha

Legumes <5ha 5–10 11–20 21–50 >50ha

Oilseeds <5ha 5–10 11–20 21–50 >50ha

Sugar beets / Fodder beets / Potatoes <5ha 5–10 11–20 21–50 >50ha

Vegetables <5ha 5–10 11–20 21–50 >50ha

Fruit / Stone fruit <5ha 5–10 11–20 21–50 >50ha

Berries <5ha 5–10 11–20 21–50 >50ha

Grapevines <5ha 5–10 11–20 21–50 >50ha

Forest <5ha 5–10 11–20 21–50 >50ha

Other, please specify: ______________ <5ha 5–10 11–20 21–50 >50ha

**4.4) What is the approximate total size of the farm?** Please select only one answer.

Less than 5 hectares/5–10 hectares/11–20 hectares/21–50 hectares/Larger than 50 hectares

**4.5) Which production methods are used on the farm?** Multiple possible answers.

Conventional/Integrated Production (IP)/Organic/Currently in conversion to organic production/Other, please specify: ___________________________

**4.6) For approximately how many years has this production method been used / has the conversion to organic farming been in progress?** Multiple possible answers.

Conventional: __________ (in full years)

Integrated Production: __________ (in full years)

Organic: __________ (in full years)

Conversion to organic production: __________ (in full years)

Other: __________ (in full years)

**4.7) What are the three main sources of income for the farm?**

*Please list three products or services that generate the highest revenues for the farm. Answers may be given in any order.*

1 _______________________________

2 _______________________________

3 _______________________________

**4.8) How many people live permanently or for an extended period in your household, including yourself?**

Number of adults (including yourself): ______

Number of persons under 18 years of age: ______

4.9) **How many people work on the farm, including yourself?**

*This includes part-time and full-time employees, seasonal workers, as well as, for example, family members who work on the farm without pay.*

Colder season, number of adults (including yourself): __________

Warmer season, number of adults (including yourself): __________

**General Health**

**5.1) How would you describe your general state of health?** Please select only one answer.

Excellent/Very good/Good/Fair/Poor

**5.2) The following questions refer to activities you might perform during a typical day. For each activity, please indicate whether your current health limits you in carrying out these activities.**

*Please select only one answer for each activity. If the examples do not apply to you, answer the question based on an activity of similar physical intensity.*

Moderate activities Yes, limited a lot Yes, limited a little No, not limited at all

(e.g., moving a table or bowling)

Climbing several flights of stairs Yes, limited a lot Yes, limited a little No, not limited at all

**5.3) During the past 4 weeks, how often have you had any of the following problems at work or in your daily activities at home due to your physical health?**

*Please select only one answer for each statement.*

I accomplished less than I would have liked. Always/Mostly/Sometimes/Rarely/Never

I was limited in the kinds of activities I could do. Always/Mostly/Sometimes/Rarely/Never

**5.4) During the past 4 weeks, how often have you had any of the following problems at work or in your daily activities at home due to emotional problems (for example, feeling depressed or anxious)?**

*Please select only one answer for each statement.*

I accomplished less than I would have liked. Always/Mostly/Sometimes/Rarely/Never

I did not work as carefully as usual. Always/Mostly/Sometimes/Rarely/Never

**5.5) To what extent did pain interfere with your normal work (including work outside the home and housework) during the past 4 weeks?** Please select only one answer.

Not at all/A little/Moderately/Quite a bit/Extremely

**5.6) These questions are about how you feel and how things have been for you during the past 4 weeks. Please choose the answer that best describes how you have felt.**

*Please select only one answer for each statement.*

During the past 4 weeks, how often have you been…

calm and relaxed? Always/Mostly/Sometimes/Rarely/Never

full of energy? Always/Mostly/Sometimes/Rarely/Never

discouraged and sad? Always/Mostly/Sometimes/Rarely/Never

**5.7) During the past 4 weeks, how often have your physical health or emotional problems interfered with your social activities (such as visiting friends or relatives)?** Please select only one answer.

Always/Mostly/Sometimes/Rarely/Never

**Physical Activity, Nutrition, Alcohol, and Tobacco**

**6.1) Approximately how many hours per day do you spend sitting?**

On a workday during the colder season: _______ (hours per day)

On a rest day during the colder season: _______ (hours per day)

On a workday during the warmer season: _______ (hours per day)

On a rest day during the warmer season: _______ (hours per day)

**6.2) On how many days per week are you physically active for at least 30 minutes in your agricultural work (active enough to make you breathe harder or sweat)?** Please select only one answer.

If you do not work on a farm, please proceed directly to Question 6.2.1.

Colder season 0/1/2/3/4/5/6/7 days per week

Warmer season 0/1/2/3/4/5/6/7 days per week

**6.2.1) If you do not work on a farm: On how many days per week are you physically active for at least 30 minutes in your occupation (active enough to make you breathe harder or sweat)?** Please select only one answer.

If you work on a farm, please proceed directly to Question 6.3.

Colder season 0/1/2/3/4/5/6/7 days per week

Warmer season 0/1/2/3/4/5/6/7 days per week

I am not employed

**6.3) On how many days per week are you physically active for at least 30 minutes during your leisure time (active enough to make you breathe harder or sweat)?** Please select only one answer.

Colder season 0/1/2/3/4/5/6/7 days per week

Warmer season 0/1/2/3/4/5/6/7 days per week

**6.4) On how many days per week do you usually eat the following foods?** Please select only one answer for each item.

Red meat or sausage/cold cuts Never/Less than 1 day/1/2/3/4/5/6/7/Don’t know

Cooked vegetables Never/Less than 1 day/1/2/3/4/5/6/7/Don’t know

Raw vegetables or salad Never/Less than 1 day/1/2/3/4/5/6/7/Don’t know

Fruit Never/Less than 1 day/1/2/3/4/5/6/7/Don’t know

**6.5) When you eat vegetables or fruit, how many portions do you usually consume per day on average?** Please select only one answer for each item.

*One portion corresponds approximately to a handful or one large tomato.*

Raw vegetables or salad <1 portion/1/2–4/5+ portions/Don’t know

Cooked vegetables <1 portion/1/2–4/5+ portions/Don’t know

Fruit <1 portion/1/2–4/5+ portions/Don’t know

**6.6) How often do you consume the following alcoholic beverages?** Please select only one answer for each item.

Beer / Wine / Never/1–4x per month or less/Several times per week/Daily

Sparkling wine / Cider

Spirits / Liqueur Never/1–4x per month or less/Several times per week/Daily

**6.7) Do you use any of the following tobacco products?** Please select only one answer for each item.

Cigarettes Never smoked/Formerly/Yes, on some days/Yes, daily

E-cigarettes Never smoked/Formerly/Yes, on some days/Yes, daily

Other tobacco products Never smoked/Formerly/Yes, on some days/Yes, daily

**6.8) Approximately how much do you weigh?** Please specify whether this weight is self-reported (estimated) or measured.

*For women who are currently pregnant: please indicate your approximate weight at the beginning of your pregnancy.*

______ kg estimated/measured

**6.9) Approximately how tall are you?**

**______ cm**

**Your Assessment of Occupational Risks**

**If you do not work on a farm yourself, please skip all questions in the section “Your Assessment of Occupational Risks” and proceed directly to the section “Quality of Life, Sleep, and Stress.”**

In agricultural health research, five main areas of occupational hazards are generally distinguished:

- Physical (e.g., prolonged sitting, manual labor)
- Chemical (e.g., fertilizers, pesticides, gases, and other hazardous substances)
- Biological (e.g., infectious diseases, pollen, dust)
- Psychosocial (e.g., social isolation, conflicts/disputes, stress/pressure)
- Environmental (e.g., storms, heat/cold, landslides)

We would like to know:

a) how often, on average, you have been exposed to specific hazards within each of these five areas in your agricultural work during the past 12 months, and

b) how harmful to health you consider each of these hazards to be, regardless of how often you have personally been exposed to them.

For each area, four example activities (hazards) are listed. In the final blank row, you may add another hazard that you think should be mentioned, and mark the corresponding boxes.

“Never” means that such activities are performed on the farm but not by you, or that other people are exposed to these hazards, but not you.

“Not relevant” means that no one on the farm performs such activities or is exposed to such hazards.

**7.1) Physical hazards: During the past 12 months, how often have you performed the following activities in your agricultural work? Regardless of how often you performed them, how harmful to health do you consider each activity to be?**

*Scale for each hazard:*

*Frequency: Never/ Rarely/Occasionally/Often/Always/Not relevant*

*Harmfulness: Not health-damaging/Slightly/Moderately/Quite/Very*

Heavy physical work (e.g., carrying loads, manual mowing)

Operating vehicles or machinery

Working at heights or on difficult terrain (e.g., on a ladder, roof, or slope)

Prolonged sitting (4 or more hours at a time)

**7.2) Chemical hazards: On average, how often have you performed the following activities in your agricultural work during the past 12 months? Regardless of how often you performed them, how harmful to your health do you consider each activity to be?**

*Scale for each hazard:*

*Frequency: Never/ Rarely/Occasionally/Often/Always/Not relevant*

*Harmfulness: Not health-damaging/Slightly/Moderately/Quite/Very*

Application of fertilizers

Mixing and/or applying plant protection products (e.g., herbicides, fungicides, or insecticides)

Activities involving exposure to gases or fumes (e.g., from manure)

Use of corrosive cleaning agents (e.g., machinery cleaners)

**7.3) Biological hazards: On average, how often have you performed the following activities in your agricultural work during the past 12 months? Regardless of how often you performed them, how harmful to your health do you consider each activity to be?**

*Scale for each hazard:*

*Frequency: Never/ Rarely/Occasionally/Often/Always/Not relevant*

*Harmfulness: Not health-damaging/Slightly/Moderately/Quite/Very*

Pollen or spores (e.g., grass pollen or mold spores)

Dust (e.g., wood dust or hay dust)

Animals or animal excretions (e.g., direct contact with unrestrained animals, manure, or blood)

Insects that may sting or bite(e.g., bees or ticks)

**7.4) Psychosocial hazards: On average, how often have you been exposed to the following psychosocial hazards in your agricultural work during the past 12 months? Regardless of how often you were personally exposed, how harmful to your health do you consider each hazard to be?**

*Scale for each hazard:*

*Frequency: Never/ Rarely/Occasionally/Often/Always/Not relevant*

*Harmfulness: Not health-damaging/Slightly/Moderately/Quite/Very*

Conflicts (e.g., with employees, within the family, or with neighbors)

Sleep problems

Stress or pressure

Loneliness

**7.5) Environmental hazards: On average, how often have you been exposed to the following environmental hazards in your agricultural work during the past 12 months? Regardless of how often you were personally exposed, how harmful to your health do you consider each hazard to be?**

*Scale for each hazard:*

*Frequency: Never/ Rarely/Occasionally/Often/Always/Not relevant*

*Harmfulness: Not health-damaging/Slightly/Moderately/Quite/Very*

Working outdoors in direct sunlight

Storms (e.g., hail, lightning, or heavy rainfall)

Landslides or avalanches

Noise

**Quality of Life, Sleep, and Stress**

**8.1) Below you will find 12 questions and statements. Please answer each on a scale from 0 to 10. Mark only one response per question/statement.**

Overall, how satisfied are you with life as a whole these days?

0=Not Satisfied at All/1/2/3/4/5/6/7/8/9/10=Completely Satisfied

In general, how happy or unhappy do you usually feel?

0=Extremely unhappy/1/2/3/4/5/6/7/8/9/10=Extremely happy

In general, how would you rate your physical health?

0=Poor/1/2/3/4/5/6/7/8/9/10=Excellent

How would you rate your overall mental health?

0=Poor/1/2/3/4/5/6/7/8/9/10=Excellent

Overall, to what extent do you feel the things you do in your life are worthwhile?

0=Not at All Worthwhile/1/2/3/4/5/6/7/8/9/10=Completely Worthwhile

I understand my purpose in life.

0=Strongly disagree/1/2/3/4/5/6/7/8/9/10=Strongly agree

I always act to promote good in all circumstances, even in difficult and challenging situations.

0=Not true of me/1/2/3/4/5/6/7/8/9/10=Completely true of me

I am always able to give up some happiness now for greater happiness later.

0=Not true of me/1/2/3/4/5/6/7/8/9/10=Completely true of me

I am content with my friendships and relationships.

0=Strongly disagree/1/2/3/4/5/6/7/8/9/10=Strongly agree

My relationships are as satisfying as I would want them to be.

0=Strongly disagree/1/2/3/4/5/6/7/8/9/10=Strongly agree

How often do you worry about being able to meet normal monthly living expenses?

0=Worry all of the time/1/2/3/4/5/6/7/8/9/10=Do not ever worry

How often do you worry about safety, food, or housing?

0=Worry all of the time/1/2/3/4/5/6/7/8/9/10=Do not ever worry

**8.2) How many hours per night did you sleep on average during the past 12 months? This does not have to correspond to the number of hours you spent in bed.**

*Please indicate the number in whole hours.*

Colder season _______

Warmer season _______

**8.3) How would you rate the overall quality of your sleep during the past 12 months?** Please check only one answer.

Colder season Very good/good/fair/poor/very poor

Warmer season Very good/good/fair/poor/very poor

**8.4) During the past 12 months, how often did you…**

*Please check only one answer for each question.*

Have difficulty falling asleep? Never/rarely/sometimes/often

Have restless sleep? Never/rarely/sometimes/often

Wake up several times during the night? Never/rarely/sometimes/often

Wake up too early in the morning? Never/rarely/sometimes/often

**8.5) On a scale from 1 to 6, how would you rate your ability to cope with stress?**

(1 = I can shake off stress easily, 6 = stress wears me down) Please select only one answer.

1/2/3/4/5/6

**8.6) During the past 12 months, how would you rate the overall level of stress in your life (at home and at work)?** **(1 = no stress, 6 = extreme stress)** Please select only one answer.

Colder season 1/2/3/4/5/6

Warmer season 1/2/3/4/5/6

**8.7) How have the following aspects of your health changed overall during the coronavirus (COVID-19) pandemic (since March 2020)?** Please check only one answer for each statement.

Physical health Much worse/somewhat worse/no change/somewhat better/much better

Mental health Much worse/somewhat worse/no change/somewhat better/much better

Quality of life Much worse/somewhat worse/no change/somewhat better/much better

Sleep quality Much worse/somewhat worse/no change/somewhat better/much better

Healthy lifestyle Much worse/somewhat worse/no change/somewhat better/much better

(diet, physical activity, alcohol/tobacco use)

**Job Satisfaction**

**9.1)** **During the past 12 months, how satisfied were you overall with your work on the agricultural operation?** Please select only one answer.

If you do not work on a farm, please proceed directly to Question 9.1.1.

Very satisfied/quite satisfied/ Neither satisfied nor dissatisfied/quite dissatisfied/very dissatisfied

**9.1.1) If you are not working on an agricultural operation: During the past 12 months, how satisfied were you overall with your work?** Please select only one answer.

Very satisfied/quite satisfied/ Neither satisfied nor dissatisfied/quite dissatisfied/very dissatisfied

**9.2) Regardless of whether this was paid or unpaid work (e.g., household work): During the past 12 months, how often did you work…**

*Colder season*

More than 5 days per week Never/rarely/sometimes/often

More than 10 hours per day Never/rarely/sometimes/often

*Warmer season*

More than 5 days per week Never/rarely/sometimes/often

More than 10 hours per day Never/rarely/sometimes/often

**9.3) Which of the following were the three most important reasons you chose your agricultural profession?** Please select three reasons.

If you do not work on a farm, please proceed directly to the section “Illnesses and Accidents.”

Financial security/Freedom to organize work independently/Sense of purpose or meaningfulness of the profession/Social relationships with employees, acquaintances, family, or neighbors/Flexible working hours/Social recognition/Physical work and working outdoors in nature/Job security/ Other, please specify:_____________________/Don’t know

**9.4) If you were to decide to give up your agricultural profession: Which of the following would be the three most likely reasons for doing so?** Please select three reasons.

Financial pressure/Too little free time or vacation/Lack of social recognition/Loneliness/Conflicts at work, within the family, or with neighbors/Political or social pressure/Health problems/Irregular or long working hours/Difficulty finding a successor for the farm/Other, please specify: ___________________________/Don’t know

**Illnesses and Accidents**

**All information will be treated strictly confidentially.**

**10.1) Has a doctor ever told you that you have (or have had) any of the following conditions?** Please check only one answer for each condition.

*If you have received a medical diagnosis for a condition, please also answer the follow-up questions.*

*Response options for each condition: Yes/no/don’t know/no response*

Back pain lasting 3 months or longer and occurring almost daily

Osteoarthritis or joint degeneration

Asthma

Chronic bronchitis or chronic obstructive pulmonary disease (COPD)

Allergies (e.g., hay fever, food allergy, eczema, insect venom allergy, etc.)

Cardiovascular disease (e.g., heart attack, heart failure, cardiac arrhythmia, etc.)

Diabetes mellitus (including diabetes diagnosed during pregnancy)

Cancer (If yes: What type of cancer? ___________________________)

Stroke

Parkinson’s disease (also known as shaking palsy)

Depression or anxiety disorder

Accident/injury/fall requiring a doctor’s visit (If yes: Did this occur at work or during leisure time?)

*Follow-up questions if yes:*

In which year was this condition first diagnosed (approximately)? ________

Do you regularly take prescription medication for this condition or these symptoms? Yes/no

**10.2) How often have you been in the following places during the past 12 months?** A transfer from one hospital to another counts as one visit. If you have not been to any of these places in the past 12 months, please write 0.

At a doctor’s office/general practitioner for an outpatient visit _____/Don’t know

At a hospital or specialized clinic (without overnight stay) _____/Don’t know

At a hospital or specialized clinic (with overnight stay) _____/Don’t know

At a medical emergency department _____/Don’t know

Other, please specify: ***___________________________*** _____/Don’t know

**Final Question**

**11.1) May we contact you again in the future for a health survey or study (e.g., a follow-up health questionnaire)?**

Yes/no

**Your Opinion Matters**

**12.1) If you would like to share anything with us, please use the space below for suggestions, comments, wishes, or feedback. Your opinion is important to us.**

## S2 First Follow-up Questionnaire in English (not used in data collection)

***NOTE: The questionnaire was originally administered in German, French, and Italian. An English translation in a simple layout is provided here for the purpose of transparency and review only. The English version was not used in data collection.***

**FarmCoSwiss: Follow-up Survey on the Health of Individuals and Their Partners in Swiss Agriculture**

Dear Participant,

The Swiss Tropical and Public Health Institute (Swiss TPH) is conducting this second questionnaire to take a closer look at the general health and well-being of people working in Swiss agriculture and their spouses or partners.

This questionnaire includes both familiar and new questions. Some questions are repeated exactly as before in order to assess potential changes in your health or working conditions over time (e.g., due to seasonal differences). Based on the feedback received from the first questionnaire (thank you again for your valuable contribution!), a few new questions have also been added.

Questions marked with a **gray box** are to be completed by all participants.

Questions marked with an **orange box** are intended for participants who work in agriculture.

By taking part again, you contribute to the understanding and improvement the health of the agricultural population in Switzerland.

We take data protection and confidentiality very seriously. All information collected will be treated with **strict confidentiality**, analyzed in **encrypted** form, and used exclusively for scientific purposes.

If you have any questions or uncertainties, you can contact the study team at 061 284 89 29 (German and English) or farmcoswiss@swisstph.ch

Thank you very much for your participation!

Prof. Dr. Nicole Probst-Hensch

**Paper Questionnaire**

Please complete the questionnaire using a black or blue ballpoint pen. When finished, kindly return the completed questionnaire in the enclosed pre-addressed and prepaid envelope to the Swiss Tropical and Public Health Institute.

Thank you very much!

**Introductory Questions**

**0) Please indicate the date on which you are completing this questionnaire.**

___/___/_____ (DD-MM-YYYY)

**0.1) Do you work on a farm?**

*By "work," we mean individuals who are self-employed, employed (full-time or part-time), in training, doing seasonal work, or volunteering on a farm (e.g., as family members). Activities on the farm may include agricultural as well as administrative tasks.*

Yes/No

**0.2) Does your spouse/partner also participate in this survey?**

Yes/No/I don’t know/I don’t have a spouse or partner

**0.2.1) If your partner is also participating in the survey, please provide their full name and date of birth. We require this information in order to link you and your partner together.**

First name ________________

Last name ________________

Date of birth ________________

**0.3) If you work on a farm: Are there other people who work on the same farm as you who are also participating in this survey (excluding your spouse/partner)?**

If “no” or “don’t know”: Please proceed directly to Question 0.4.

Yes/No/I don’t know

If yes: How many people? ______

**0.3.1) If other individuals from the same agricultural operation are also participating in the survey, please provide their first and last names**. This information is needed to link you and your coworkers to one another.

Person 1: first and last name ___________________________________________________

Person 2: first and last name ___________________________________________________

Person 3: first and last name ___________________________________________________

**0.4) What is your marital status?**

Single/married or in a registered partnership/divorced/widowed

**In this questionnaire, some questions refer to the agricultural operation where you work. Please answer these questions based on the operation where you (or your partner) spent most of your working time during the past 12 months.**

**Contact Information**

**1.1) Please provide your first and last name.**

First name: ___________________

Last name: ___________________

**1.2) Have your residential and/or farm address, as provided during the last survey, changed since then?**

Yes/No

If yes, new residential address: ____________________________________

From what date is this new residential address valid? ___/___/_____ (DD-MM-YYYY)

If yes, new farm address: ____________________________________

From what date is this new farm address valid? ___/___/_____ (DD-MM-YYYY)

**1.3) Have your phone number and/or email address, as provided during the last survey, changed since then?**

Yes/No

If yes, new phone number: ________________________

If yes, new email address: ________________________

**Employment**

**2.1) What is your professional position on the farm?** Multiple possible answers.

Farm operator or co-operator (farm management)/Working in the family farm (with income subject to social security contributions)/Working in the family farm (without income subject to social security contributions)/Employee with supervisory responsibilities (managing staff)/Employee without supervisory responsibilities (no staff management)/Retired but still working/In training/Other, please specify: ___________________________

**2.2) For which areas of activity are you mainly responsible during a typical work week?** Multiple possible answers.

Administration/Off-farm employment (secondary or non-agricultural work)/Farm work/Child-rearing and childcare/Gardening and outdoor maintenance/Household work/Farm-related activities (e.g., direct marketing or agritourism)/Care of family members/Other, please specify: _______________________________________________

**Farm Management and Household Structure**

**3.1) Is the farm leased?**

Yes/no/don’t know

**3.1.1) If leased: Who is the lessor (the person/institution renting out the farm)?**

Family member/Non-relative/Public or private institution (e.g., municipality or bank)/Other, please specify: ___________________________________________

**3.2) In which region is the farm located?**

Valley region/Hilly region/Mountain region/Don’t know/Other, please specify: __________________

**3.3) What is the farm’s main production focus?** Multiple possible answers.

Livestock farming – dairy production/Livestock farming – meat production/Intensive production – pig farming/Intensive production – poultry farming/Other animal production/Arable (crop) farm/Specialized crop production (e.g. fruit, vegetables, berries, or vines)/Horticulture/ Forestry/Other plant production/Other, please specify: ________________________________

**3.4) Which production methods are used on the farm?** Multiple possible answers.

Ecological Performance Record (ÖLN)/Integrated Production (IP)/organic/biodynamic agriculture/regenerative agriculture/other, please specify: ________________________________

**3.5) Do you produce exclusively for the Swiss market or do you also export your products abroad?** Multiple possible answers.

Exclusively for the Swiss market/exclusively for export/for both the Swiss market and export/production for own use/don’t know

**3.6) Do you sell your products under one or more of the following labels?** Multiple possible answers.

Agri Natura/ Alnatura/ AOP/IGP/ Bio Suisse “Knospe”/Aus der Region. Für die Region. (Migros)/ Bio Natur Plus (Manor)/Bio Organic (Lidl)/Bio Organic Weiderind (Lidl)/Bio Weide-Beef (Migros)/ Coop Naturafarm/Coop Naturaplan/Delinat/Demeter/EU-Bio/Fidelio/KAGfreiland/Hochstamm Suisse/IP-SUISSE/Miini Region (Coop)/Migros Bio/Natura-Beef/Natura-Veal/NatureActive Bio (Aldi)/Natur pur (Spar)/Nature Suisse (Aldi)/Nature Suisse Bio (Aldi)/Pro Montagna/ProSpecieRara/regio.garantie/ retour aux sources (Aldi)/Silvestri Bio-Weiderind/Silvestri Freilandschwein/Silvestri Milchkalb/ Silvestri Weiderind/Suisse Garantie/swissmilk green/SwissPrimGourmet/Terra Natura (Lidl)/UrDinkel/ VITISWISS/VINATURA/ Weide-Beef (Migros)/No labels/don’t know/other, please specify: __________________________________

**General Health**

**4.1) How would you describe your general state of health?** Please select only one answer.

Excellent/Very good/Good/Fair/Poor

**4.2) The following questions refer to activities you might perform during a typical day. For each activity, please indicate whether your current health limits you in carrying out these activities.**

*Please select only one answer for each activity. If the examples do not apply to you, answer the question based on an activity of similar physical intensity.*

Moderate activities Yes, limited a lot Yes, limited a little No, not limited at all

(e.g., moving a table or bowling)

Climbing several flights of stairs Yes, limited a lot Yes, limited a little No, not limited at all

**4.3) During the past 4 weeks, how often have you had any of the following problems at work or in your daily activities at home due to your physical health?**

*Please select only one answer for each statement.*

I accomplished less than I would have liked. Always/Mostly/Sometimes/Rarely/Never

I was limited in the kinds of activities I could do. Always/Mostly/Sometimes/Rarely/Never

**4.4) During the past 4 weeks, how often have you had any of the following problems at work or in your daily activities at home due to emotional problems (for example, feeling depressed or anxious)?**

*Please select only one answer for each statement.*

I accomplished less than I would have liked. Always/Mostly/Sometimes/Rarely/Never

I did not work as carefully as usual. Always/Mostly/Sometimes/Rarely/Never

**4.5) To what extent did pain interfere with your normal work (including work outside the home and housework) during the past 4 weeks?** Please select only one answer.

Not at all/A little/Moderately/Quite a bit/Extremely

**4.6) These questions are about how you feel and how things have been for you during the past 4 weeks. Please choose the answer that best describes how you have felt.**

*Please select only one answer for each statement.*

During the past 4 weeks, how often have you been…

calm and relaxed? Always/Mostly/Sometimes/Rarely/Never

full of energy? Always/Mostly/Sometimes/Rarely/Never

discouraged and sad? Always/Mostly/Sometimes/Rarely/Never

**4.7) During the past 4 weeks, how often have your physical health or emotional problems interfered with your social activities (such as visiting friends or relatives)?** Please select only one answer.

Always/Mostly/Sometimes/Rarely/Never

**4.8) How often have they been bothered by the following over the past 2 weeks?** Please select only one answer for each statement.

*Scale for each statement: Not at all/several days/more than half the days/nearly every day*

Little interest or pleasure in doing things?

Feeling down, depressed, or hopeless?

Trouble falling or staying asleep, or sleeping too much?

Feeling tired or having little energy?

Poor appetite or overeating?

Feeling bad about yourself — or that you are a failure or have let yourself or your family down?

Trouble concentrating on things, such as reading the newspaper or watching television?

Moving or speaking so slowly that other people could have noticed? Or so fidgety or restless that you have been moving a lot more than usual?

Thoughts that you would be better off dead, or thoughts of hurting yourself in some way?

**4.9) How often have they been bothered by the following over the past 2 weeks?**

*Scale for each statement: Not at all/several days/more than half the days/nearly every day*

Feeling nervous, anxious, or on edge

Not being able to stop or control worrying

Worrying too much about different things

Trouble relaxing

Being so restless that it's hard to sit still

Becoming easily annoyed or irritable

Feeling afraid as if something awful might happen

**4.10) The next questions are about how you are currently feeling.** Please select only one answer for each statement.

How often do you feel that you lack companionship? Hardly ever or never/sometimes/often

How often do you feel left out? Hardly ever or never/sometimes/often

How often do you feel isolated from others? Hardly ever or never/sometimes/often

**4.11) Are you currently in a (acute) special life situation that strongly affects your general health and/or well-being?** Multiple possible answers.

No/Yes, pregnancy/Yes, illness or injury/Yes, difficult financial situation/Yes, difficult social or family situation/Yes, other (please specify): __________________________________________

**Physical Activity**

**5.1) On how many days per week are you physically active for at least 30 minutes in your (agricultural) work (active enough to make you breathe harder or sweat)?** Please select only one answer.

Colder season 0/1/2/3/4/5/6/7 days per week

Warmer season 0/1/2/3/4/5/6/7 days per week

**5.2) On how many days per week are you physically active for at least 30 minutes during your leisure time (active enough to make you breathe harder or sweat)?** Please select only one answer.

Colder season 0/1/2/3/4/5/6/7 days per week

Warmer season 0/1/2/3/4/5/6/7 days per week

**5.3) On how many of the last 7 days did you engage in vigorous physical activity for 10 minutes or longer?** (This includes activities such as heavy lifting, strenuous gardening, aerobics, or fast cycling.) Please select only one answer.

0/1/2/3/4/5/6/7

**5.3.1) If you selected any number between 1 and 7 above: How much time did you usually spend doing vigorous physical activity on one of those days?** Please select only one answer.

Less than 75 minutes (1 hour 15 minutes)/75–150 minutes (1 hour 15 minutes – 2 hours 30 minutes)/more than 150 minutes (more than 2 hours 30 minutes)/don’t know

**5.4) On how many of the last 7 days did you engage in moderate physical activity for 10 minutes or longer?** (This includes activities such as carrying light loads, light gardening, tennis, or cycling at a steady pace.) Please select only one answer.

0/1/2/3/4/5/6/7

5.4.1) **If you selected any number between 1 and 7 above: How much time did you usually spend doing moderate physical activity on one of those days?** Please select only one answer.

Less than 150 minutes (2 hours 30 minutes)/150–300 minutes (2 hours 30 minutes – 5 hours)/more than 300 minutes (more than 5 hours)/don’t know

 **If you do not work on a farm, please skip Chapters 6, 7, and 8 and go directly to Chapter 9: “Illnesses and Accidents.”**

**Assessment of Occupational Risks**

In agricultural health research, five main areas of occupational hazards are generally distinguished:

- Physical (e.g., prolonged sitting, manual labor)
- Chemical (e.g., fertilizers, pesticides, gases, and other hazardous substances)
- Biological (e.g., infectious diseases, pollen, dust)
- Psychosocial (e.g., social isolation, conflicts/disputes, stress/pressure)
- Environmental (e.g., storms, heat/cold, landslides)

In the following questions, four activities per risk area are listed that may pose health risks.

We would like to know how often, on average, you have been exposed to these activities (potential risks) in your agricultural work over the past 12 months.

“Never” means that these activities take place on the farm but not by you personally, or that other people are exposed to these risks but not you.

“Not relevant” means that these activities are not carried out on the farm, or nobody is exposed to these risks.

Additionally, we ask you how willing you are to take health risks associated with each activity. For activities you marked as “Not relevant,” please leave this question blank.

**6.1) Physical hazards: During the past 12 months, how often have you performed the following activities in your agricultural work? How willing are you to take health risks in the individual areas? (0 = not willing to take any risks at all, 10 = very willing to take risks)?**

*Scale for each hazard:*

*Frequency: Never/ Rarely/Occasionally/Often/Always/Not relevant*

*Risk-taking: 0/1/2/3/4/5/6/7/8/9/10*

Heavy physical work (e.g., carrying loads, manual mowing)

Operating vehicles or machinery

Working at heights or on difficult terrain (e.g., on a ladder, roof, or slope)

Prolonged sitting (4 or more hours at a time)

**6.2) Chemical hazards: On average, how often have you performed the following activities in your agricultural work during the past 12 months? How willing are you to take health risks in the individual areas? (0 = not willing to take any risks at all, 10 = very willing to take risks)?**

*Scale for each hazard:*

*Frequency: Never/ Rarely/Occasionally/Often/Always/Not relevant*

*Risk-taking: 0/1/2/3/4/5/6/7/8/9/10*

Application of fertilizers

Mixing and/or applying plant protection products (e.g., herbicides, fungicides, or insecticides)

Activities involving exposure to gases or fumes (e.g., from manure)

Use of corrosive cleaning agents (e.g., machinery cleaners)

**6.3) Biological hazards: On average, how often have you performed the following activities in your agricultural work during the past 12 months? How willing are you to take health risks in the individual areas? (0 = not willing to take any risks at all, 10 = very willing to take risks)?**

*Scale for each hazard:*

*Frequency: Never/ Rarely/Occasionally/Often/Always/Not relevant*

*Risk-taking: 0/1/2/3/4/5/6/7/8/9/10*

Pollen or spores (e.g., grass pollen or mold spores)

Dust (e.g., wood dust or hay dust)

Animals or animal excretions (e.g., direct contact with unrestrained animals, manure, or blood)

Insects that may sting or bite(e.g., bees or ticks)

**6.4) Psychosocial hazards: On average, how often have you been exposed to the following psychosocial hazards in your agricultural work during the past 12 months? How willing are you to take health risks in the individual areas? (0 = I strongly avoid it, 10 = I don’t avoid it at all)**

*Scale for each hazard:*

*Frequency: Never/ Rarely/Occasionally/Often/Always/Not relevant*

*Risk-taking: 0/1/2/3/4/5/6/7/8/9/10*

Conflicts (e.g., with employees, within the family, or with neighbors)

Sleep problems

Stress or pressure

Loneliness

**6.5) Environmental hazards: On average, how often have you been exposed to the following environmental hazards in your agricultural work during the past 12 months? How willing are you to take health risks in the individual areas? (0 = I strongly avoid it, 10 = I don’t avoid it at all)**

*Scale for each hazard:*

*Frequency: Never/ Rarely/Occasionally/Often/Always/Not relevant*

*Risk-taking: 0/1/2/3/4/5/6/7/8/9/10*

Working outdoors in direct sunlight

Storms (e.g., hail, lightning, or heavy rainfall)

Landslides or avalanches

Noise

**Goal Prioritization**

We would like to ask you how important you consider the improvement of certain goals in the areas of health protection, environmental protection, agricultural economics, and social policy, using pest control as an example. There are no right or wrong answers.

**7.1) How important is the improvement of each of these goals in the field of pest control to you, on a continuous scale from 0 to 100?** Please rate each goal with a whole number between 0 (improvement of the goal is completely unimportant) and 100 (improvement of the goal is extremely important).

HEALTH GOALS – HIGH HEALTH PROTECTION

High protection for users, subsequent workers, and bystanders, i.e., low acute toxicity of pest control agents and low direct exposure of users, subsequent workers, and bystanders __________

High protection for consumers, i.e., low chronic toxicity of pest control agents and low intake of (various) pest control agents through consumption of agricultural products ___________

Low workload in agriculture, i.e., low psychological (e.g., well-being) and physical strain (e.g., ratio of required vs. available workforce) for persons working in agriculture __________

ENVIRONMENTAL GOALS – HIGH ENVIRONMENTAL PROTECTION

High protection of non-target organisms, i.e., high diversity and abundance of terrestrial species on and around agricultural farms __________

High soil protection, i.e., low land consumption and high soil fertility (low soil compaction/erosion and high microbial activity) __________

High climate protection, i.e., low greenhouse gas emissions and low energy consumption for food production to meet national demand __________

High water protection, i.e., strong protection of aquatic organisms and high quality of surface water, groundwater, and drinking water __________

AGRICULTURAL ECONOMIC GOALS – HIGH AGRICULTURAL ECONOMIC POTENTIAL

High autonomy of farmers, i.e., high economic independence and high operational decision-making freedom __________

High food security, i.e., high contribution of domestic production to meet the national food demand for agricultural products __________

Low food costs, i.e., low costs of food for consumers relative to their household budget __________

High profitability, i.e., high value creation and low expenses (investment and operating costs) for farmers __________

SOCIO-POLITICAL GOALS – HIGH SOCIO-POLITICAL POTENTIAL

High cost fairness, i.e., low external costs borne by the public and high cost transparency according to the polluter pays principle __________

High international coherence, i.e., high compliance with international law and international standards __________

High innovation potential, i.e., rapid adaptability of national food production to new findings (e.g., on pest control risks) and situations (e.g., climate change) __________

High job security, i.e., preservation or creation of jobs and farms in rural areas and agriculture __________

High landscape quality, i.e., positive perception of the appearance of the cultural landscape (e.g., agricultural infrastructure like greenhouses) __________

**Health Protection**

**8.1) In general, how do you protect your physical health in your daily agricultural work**? Multiple possible answers.

Complying with legal requirements (e.g., road traffic regulations)/Acquiring (new) knowledge (e.g., about safe working techniques)/Using personal protective equipment (e.g., gloves, helmet, hearing protection)/Regular maintenance of machines or tools/Regular breaks from physically demanding and/or monotonous tasks/Regular breaks from sedentary activities (e.g., standing or walking) /Regular physical activity/I do not actively or consciously protect my physical health/Other, please specify: ________________________________________

**8.2) In general, how do you protect your mental/psychological health in your daily agricultural work?** Multiple possible answers.

Acquiring (new) knowledge (e.g., about stress management)/Careful or gentle work (e.g., avoiding time pressure or stress)/Regular rest/recovery times (e.g., hobbies, time with friends or family)/Regular holidays/Regular physical activity/Relaxation exercises (e.g., meditation, praying, or journaling)/Conversations with trusted persons (e.g., friends)/I do not actively or consciously protect my mental health/Other, please specify: ________________________________________

**8.3) Where do you get or look for information regarding health protection and accident prevention in agriculture (e.g., about personal protective equipment or safe handling of machinery)?** Multiple possible answers.

Mandatory courses or training/Voluntary courses or training/Advisory office for accident prevention in agriculture (BUL)/National information services (e.g., State Secretariat for Economic Affairs SECO or Swiss professional societies for occupational safety)/Cantonal information services (e.g., cantonal offices or competence centers)/Health insurance providers (e.g., SUVA or agrisano)/Agricultural associations (e.g., Farmers’ Association)/Agricultural events (e.g., fairs)/Agricultural magazines (e.g., SchweizerBauer)/Other farmers or colleagues/I do not look for information in the area of health protection/accident prevention/Other, please specify: ________________________________________

**8.4) What is important to you when choosing a source of information regarding health protection and accident prevention?** Multiple possible answers.

Trust in the information source/Competence of the information source/A high degree of accuracy of the information/Information can be easily applied/implemented/Previous positive experiences with the information source/The information source has similar goals as I do/Other, please specify: ________________________________________

**8.5) Which information format do you use for information regarding health protection and accident prevention?** Multiple possible answers.

Apps/Social media (e.g., Facebook)/Websites/E-mail newsletters/Messaging services (e.g., WhatsApp)/Magazines/Other printed materials (e.g., brochures)/Television or radio/Personal contact/Other, please specify: ________________________________________

**8.6) How supported and represented do you feel by regional/cantonal and national agricultural associations in the field of health protection and accident prevention in agriculture?** Please select only one answer.

Not at all/hardly/moderately/very much/completely

**Illnesses and Accidents**

**9.1) In the past 12 months, have you had a physical or mental/psychological health problem?** Multiple possible answers.

No/Yes, physical/Yes, mental

**9.1.1) If yes: In the past 12 months, did you receive treatment or professional support for this problem?** Multiple possible answers.

No/Yes, treatment or support for a physical problem/Yes, treatment or support for a mental problem

**9.2) If you received treatment or support for a physical problem: What was the profession of the person who treated/supported you?** Multiple possible answers.

General practitioner or internist/Other medical doctor/Pharmacist/Physiotherapist/Complementary medicine therapist (e.g., naturopath, magnetopath, Ayurveda practitioner)/Other non-medical therapist (e.g., dance, art, or drama therapist; yoga instructor; shaman)/Other profession: _____________________/Don’t know

**9.3) If you received treatment/support for a mental problem: What was the profession of the person who treated/supported you?** Multiple possible answers.

Psychologist, psychotherapist/Psychiatrist/General practitioner/Other medical doctor/Pharmacist/Pastor or Christian spiritual counselor/Complementary medicine therapist (e.g., naturopath, magnetopath, Ayurveda practitioner)/Other non-medical therapist (e.g., dance, art, or drama therapist; yoga instructor; shaman)/Other profession:______________________________/ Don’t know

**9.4) In the past 12 months, have you had one or more accidents that required one or more doctor visits?**

Yes/No

**9.4.1) If yes: How many accidents?**

1/2/3/4/5/More than 5

**9.4.2) If 1 or more accidents occurred: Did they happen during your free time or while performing your agricultural work/other employment?**

Accident 1 Free time/At work

Accident 2 Free time/At work

Accident 3 Free time/At work

Accident 4 Free time/At work

Accident 5 Free time/At work

**Final Question**

**10.1) May we contact you again for a future health survey or study (e.g., a follow-up health questionnaire)?**

Yes/No

**Your Opinion Matters**

**11.1) If you would like to share anything with us, you can use the space below for suggestions, wishes, comments, or criticism. Your feedback is important to us.**
